# Supplementary material for: Epigenetic control of melanoma cell invasiveness by the stem cell factor SALL4
Source: Nat Commun. 2021 Aug 20;12:5056. doi: 10.1038/s41467-021-25326-8 (PMC8379183; doi:10.1038/s41467-021-25326-8)
Supplement: Supplementary file 1 — Supplementary Information [file 41467_2021_25326_MOESM1_ESM.pdf]

Supplementary Figures and Tables

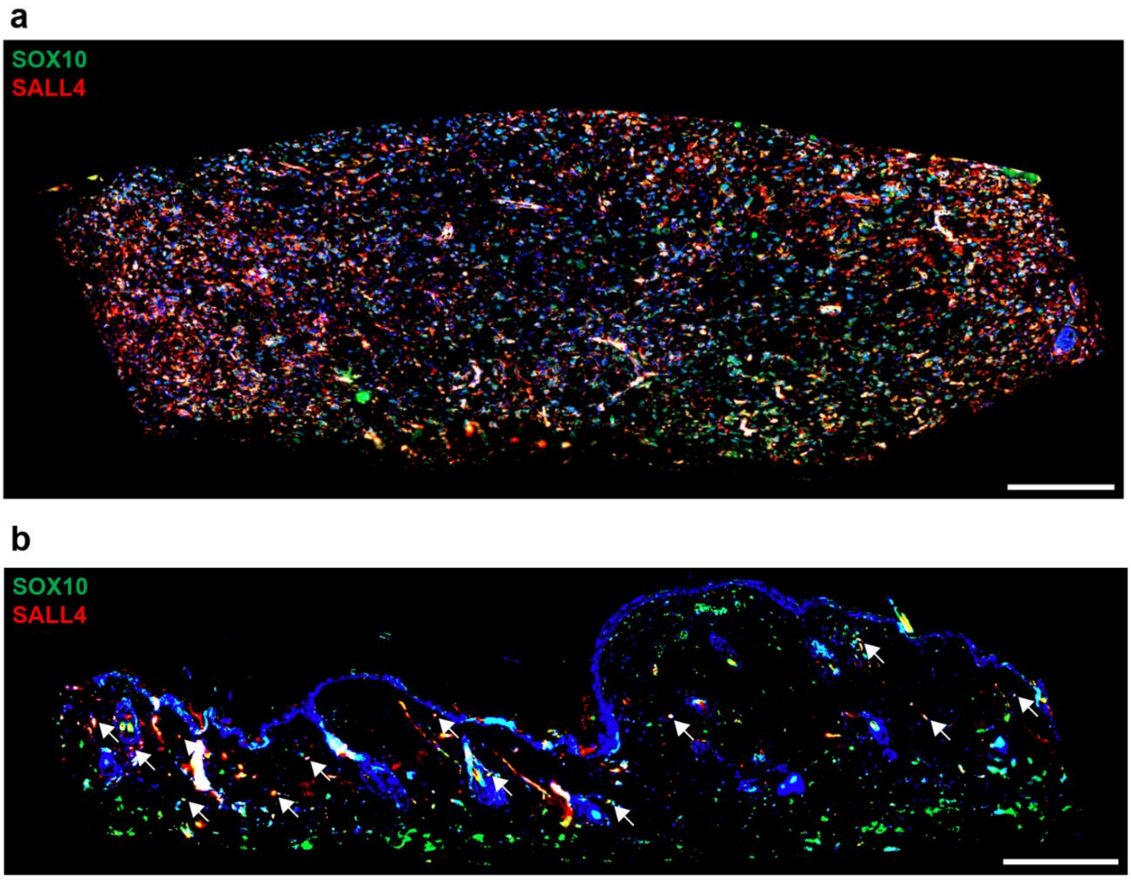

Supplementary Figure 1: Sall4 expression in murine *Tyr::Nras*^{Q61K} *Cdkn2a*$^{-/-}$ skin. **a** Immunohistochemical staining of a *Tyr::Nras*^{Q61K} *Cdkn2a*$^{-/-}$ melanoma primary tumor. **b** Immunohistochemical staining of *Tyr::Nras*^{Q61K} *Cdkn2a*$^{-/-}$ hyperplastic skin. Sox10 was used as melanocyte marker and arrows within (b) point towards melanocytes within the hyperplastic lesion that are positive for Sall4. Samples were derived from the same animal (adjacent areas on trunk back skin) and collected at an age of 5.5 month. Scale bars 200 µm. Experiments were repeated independently and with similar results three times.

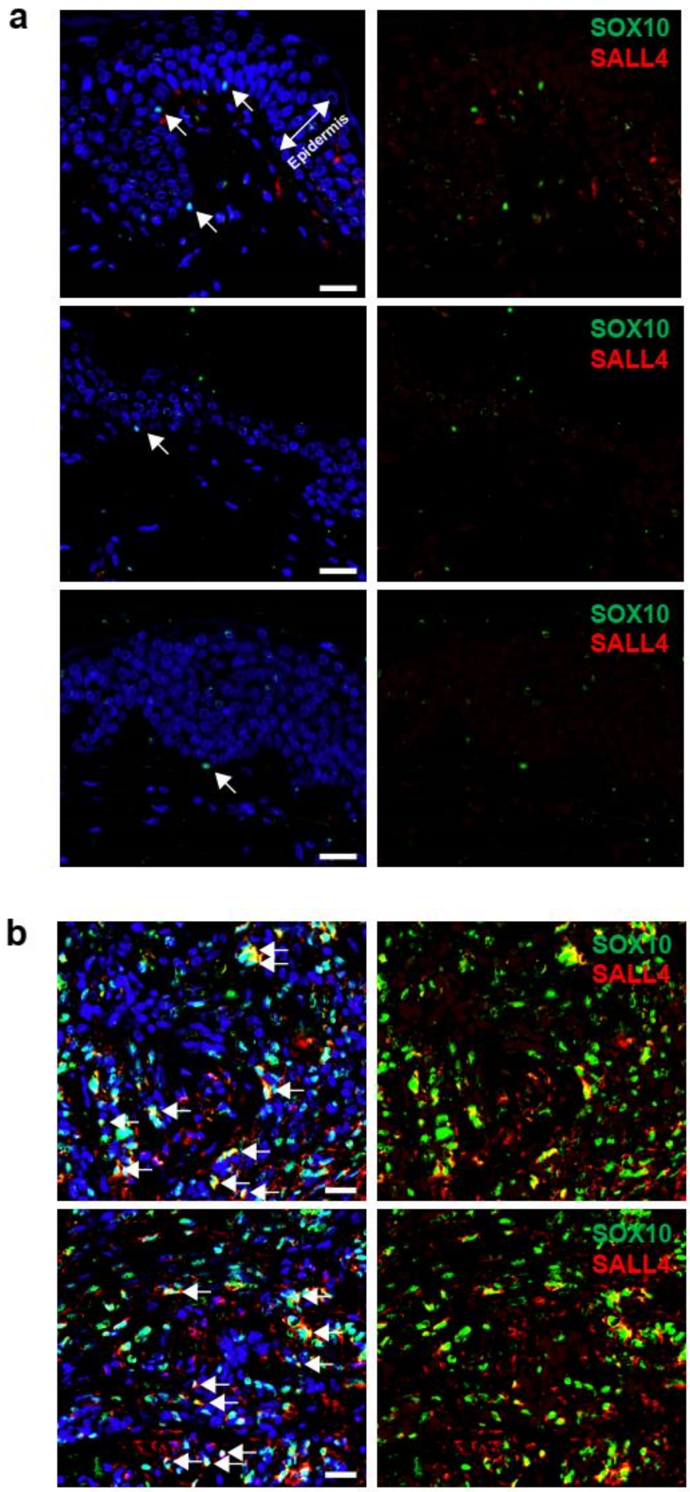

Supplementary Figure 2: SALL4 expression in human skin and melanoma. **a** Healthy human skin. **b** Human melanoma. Arrows in (a) point towards SOX10 positive melanocytes in the basal layer of the healthy human epidermis, which are SALL4 negative. Arrows in (b) point towards SOX10 positive, SALL4 positive melanoma cells within a cutaneous primary tumor. Scale bars 20 µm. Experiments were repeated independently and with similar results four times.

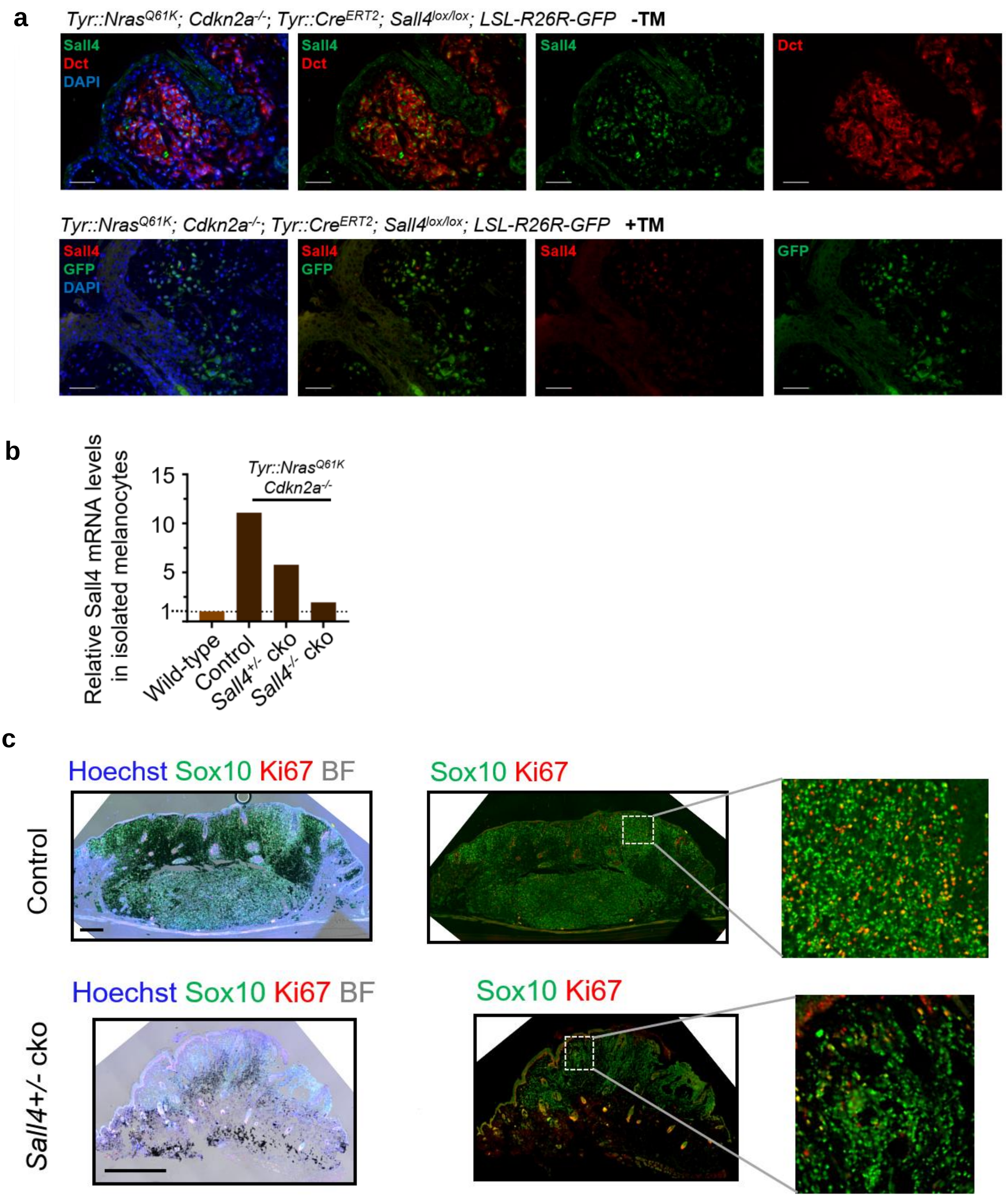

Legend on next page

Supplementary Figure 3: Sall4 loss and metastasis formation in the transgenic melanoma mouse model. **a** Immunohistochemical stainings of back skin from *Tyr::Nras*^{Q61K}*; Cdkn2a*$^{-/-}$*; Tyr::Cre*^{ERT2}*; Sall4*$^{lox/lox}$*; LSL-R26R-GFP* mice without tamoxifen (TM) injection (upper panel) and with TM injection (*Sall4*$^{-/-}$ cko) (lower panel). Dct stains melanocytes and GFP staining verifies recombination after TM injection. Scale bars 50 µm. Experiment was repeated independently and with similar results five times. **b** qRT-PCR based Sall4 expression analysis of melanocytes isolated from skin of 8 day old, TM-recombined neonatal mice of either wild-type (*Tyr::Cre*^{ERT2}*; LSL-R26R-GFP or -tdTomato*), Control *(Tyr::Nras*^{Q61K}*; Cdkn2a*$^{-/-}$*; Tyr::Cre*^{ERT2}*; Sall4*$^{wt/wt}$*; LSL-R26R-GFP or -tdTomato), Sall4*$^{+/-}$ cko *(Tyr::Nras*^{Q61K}*; Cdkn2a*$^{-/-}$*; Tyr::Cre*^{ERT2}*; Sall4*$^{wt/lox}$*; LSL-R26R-GFP or -tdTomato)* or *Sall4*$^{-/-}$ cko *(Tyr::Nras*^{Q61K}*; Cdkn2a*$^{-/-}$*; Tyr::Cre*^{ERT2}*; Sall4*$^{lox/lox}$*; LSL-R26R-GFP or -tdTomato)* genotype. Per sample, isolated melanocytes of 6-10 pups of the same genotype had to be pooled to get enough RNA for analysis by qRT-PCR. Source data are provided as Source Data file. **c** Immunohistochemical stainings of primary tumor sections from Control (*Tyr::Nras*^{Q61K}*; Cdkn2a*$^{-/-}$*; Tyr::Cre*^{ERT2}*; Sall4*$^{wt/wt}$*)* and *Sall4*$^{+/-}$ cko (*Tyr::Nras*^{Q61K}*; Cdkn2a*$^{-/-}$*; Tyr::Cre*^{ERT2}*; Sall4*$^{wt/lox}$ +TM) animals to assess proliferation rate. Sox10 was used as melanoma marker and Ki67 labels proliferating cells. These are representative images for the analysis of total 10 tumors per group quantified and presented in main Figure 2e. Scale bars: 100 µm.

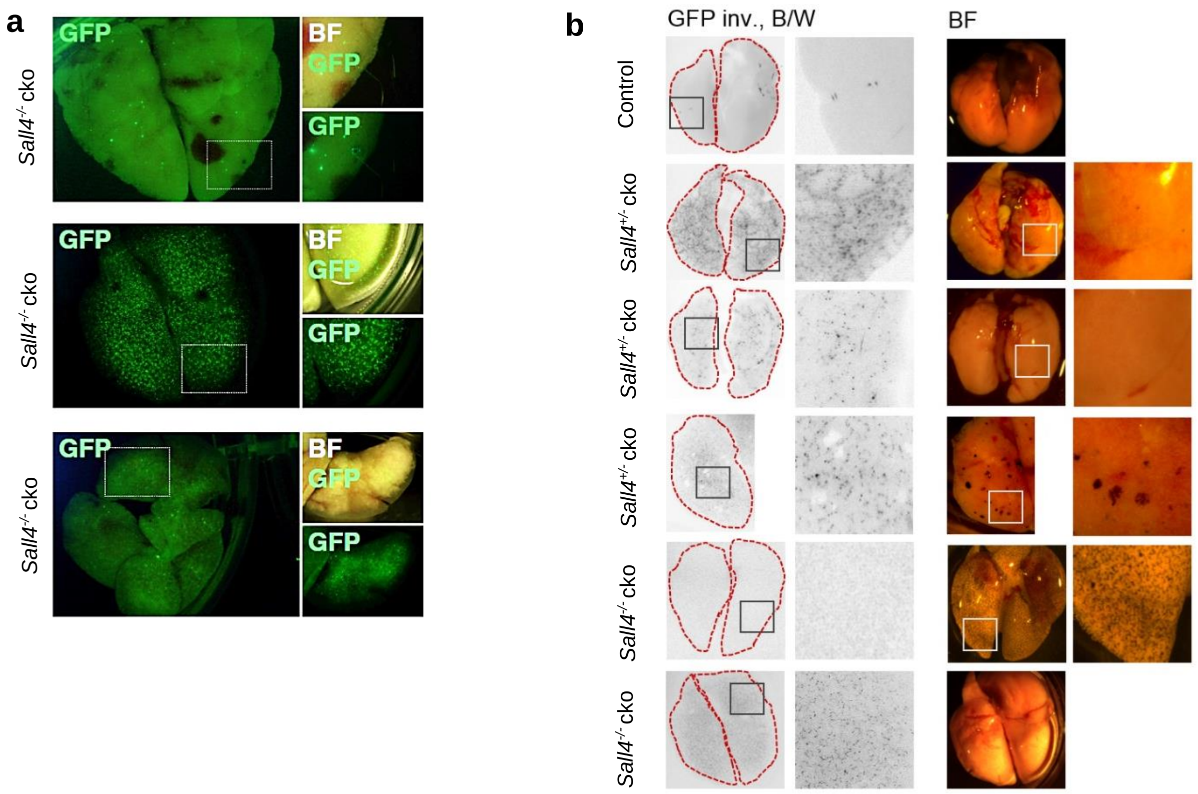

Supplementary Figure 4: Examples of endogenous GFP signal in lungs of TM-injected *Tyr::Nras*^{Q61K}; *Cdkn2a*$^{-/-}$; *Tyr::Cre*^{ERT2}; *Sall4*$^{lox/lox}$ or *Sall4*$^{lox/wt}$ (*Sall4*$^{-/-}$ cko or *Sall4*$^{+/-}$ cko) animals at termination of the experiment and assessment of metastases. **a** Examples of GFP positive micrometastases detected in lungs of *Sall4*$^{-/-}$ cko animals. Images were taken with a fluorescent binocular to detect the endogenous GFP signal. BF: bright field. **b** Example Images from lungs of *Sall4*$^{-/-}$ cko as well as *Sall4*$^{+/-}$ cko animals of endogenous GFP signal as seen in (a), inverted (inv.) and set to black and white (B/W) in ImageJ for visualization purposes. A part of this picture (left panel) is shown in main Figure 2f. Bright field (BF) images of the same lungs (right panel) show that GFP^{+} micrometastases are not always pigmented. These are representative images for the analysis of total 26 lungs for the Control group, 15 lungs for the *Sall4*$^{+/-}$ cko group and 26 lungs for the *Sall4*$^{-/-}$ cko group, which were quantified by means of GFP+ recombined metastases counts, as presented in main Figure 2h.

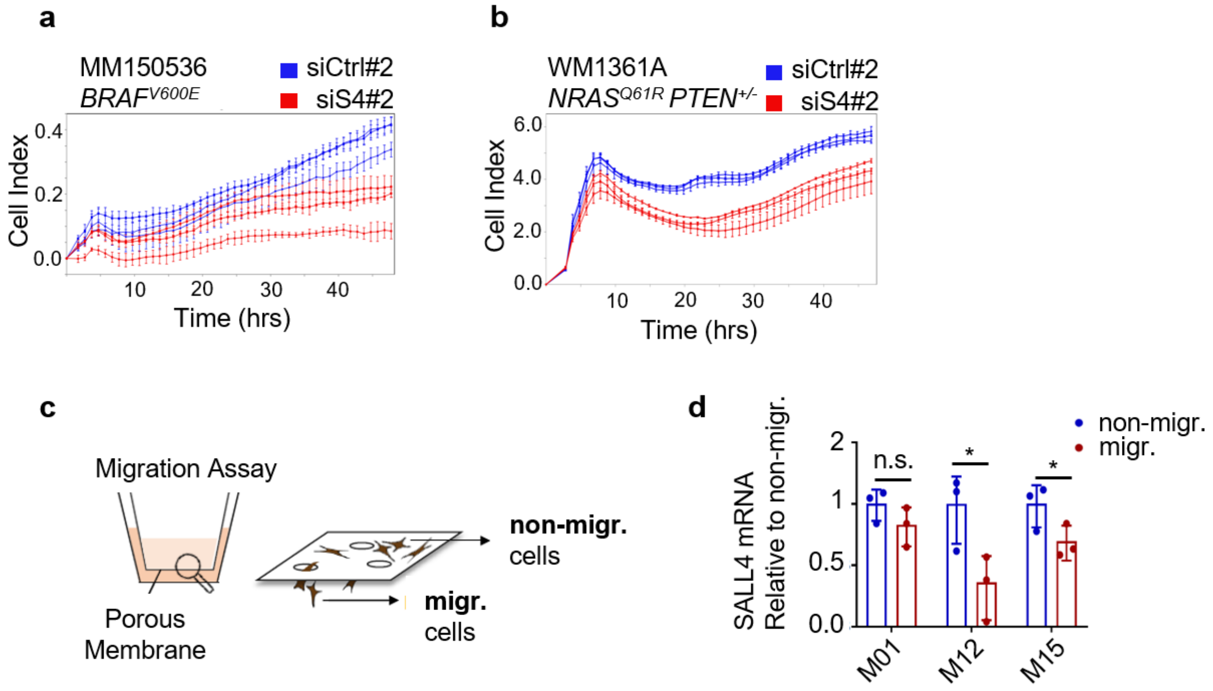

Supplementary Figure 5: SALL4 levels in human melanoma cells correlate with proliferation and anti-correlate with migration. **a-b** xCELLigence proliferation assays in additional *BRAF*^{V600E}-mutant (MM150536) and *NRAS*^{Q61R} *PTEN*$^{+/-}$-mutant (WM1361A) human melanoma cell lines. **c** Experimental Scheme of the Corning Migration Assay: non-treated cells were seeded onto the porous membrane of the well inserts and let to migrate from FCS-free (top chamber) to FCS high (lower chamber) medium for 16 hrs. Then cells on top of the membrane (non-migratory cells; non-migr.) and cells at the bottom side of the membrane that had successfully migrated through the porous membrane (migratory cells; migr.) were isolated separately to analyze cell fractions with differential migratory capacity. **d** Quantification of SALL4 expression (normalized to ACTB) in non-migratory and migratory M010817, M121224 and M150548 human melanoma cells subjected to the Corning Migration Assay as depicted in (c). Cells of three membranes were pooled to get enough material for each sample for analysis by qRT-PCR. Error bars represent mean ± SD for N = 3 with each consisting of the average of 4 technical replicas (wells measured in the xCELLigence device) in (a) and (b) and N = 3 in (d) with significance calculated by two-sided T-tests with p-values ≥ 0.05 = n.s.; < 0.05 = *; < 0.01 = ** and < 0.001 = *** with M12 in (d) P = 0.0474; M15 in (d) P = 0.0422. Source data for (a), (b) and (d) are provided as Source Data file.

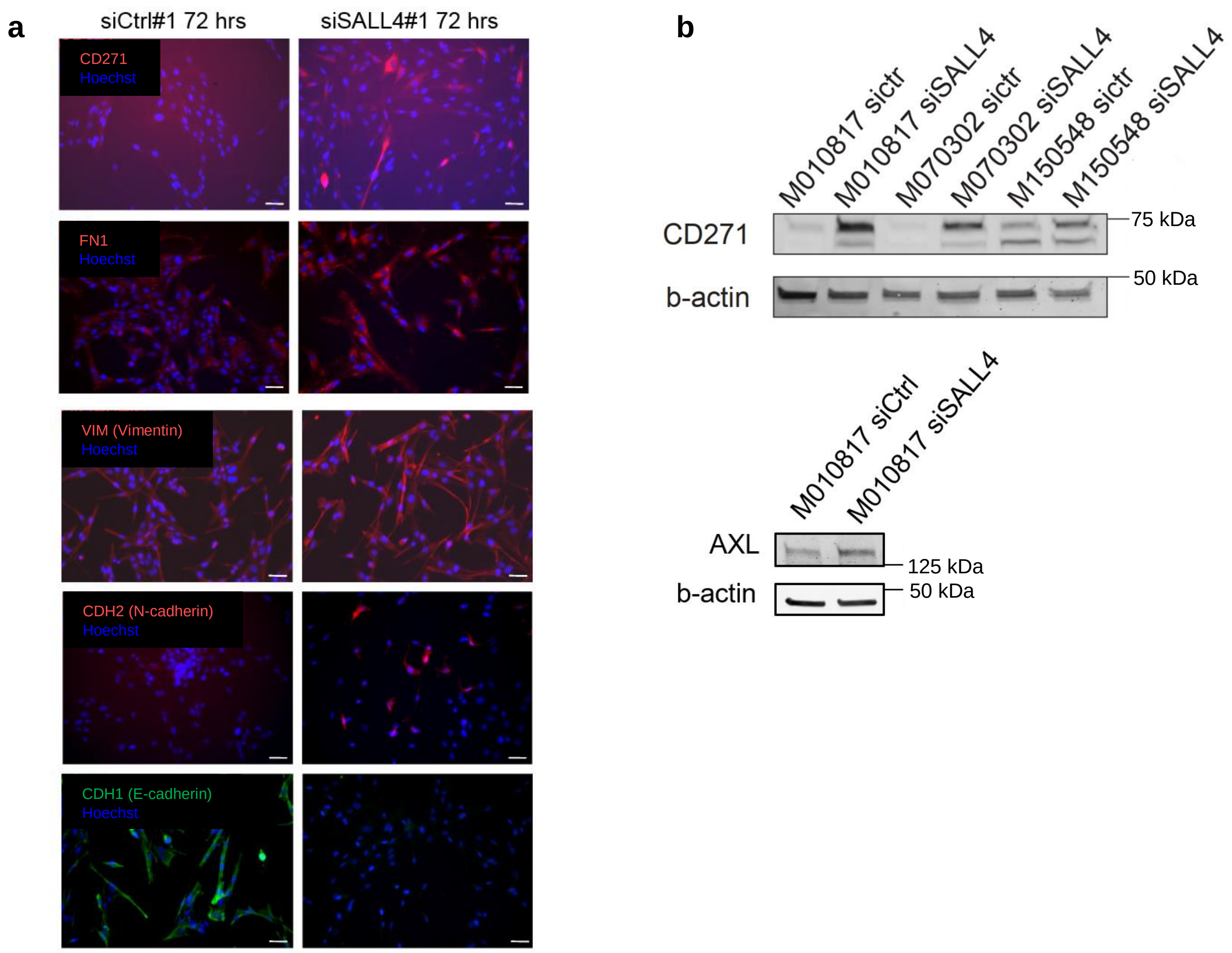

Supplementary Figure 6: Knock down of SALL4 leads to upregulation of invasiveness genes. **a** Immunocytochemistry of M010817 cells 72 hours after treatment with Control or SALL4 siRNA#1. After SALL4 knockdown, the cultured cells upregulate CD271 (NGFR), FN1, Vimentin and CDH2 (N-Cadherin), which are associated with increased melanoma invasiveness and downregulate the epithelial marker CDH1 (E-Cadherin). Scale bars 50 µm. Experiment has been repeated with similar results in one independent experiment. **b** Western blot analysis of protein levels of CD271 (NGFR) and AXL – two invasiveness genes -, which are both elevated after 72 hrs knock down with siSALL4#1 compared to siCtrl#1 on M010817 cells. Source data are provided as Source Data file. Experiment has been repeated independently two times. See also Supplementary Figure 19b for the downregulation on western blot level of the melanocyte differentiation gene MLANA after SALL4 knock down.

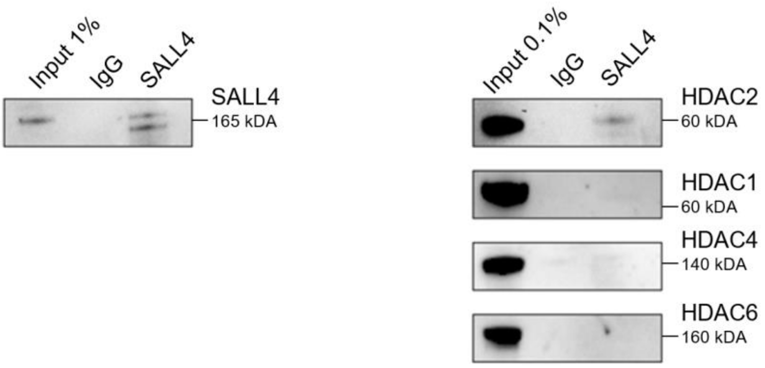

Supplementary Figure 7: Co-IP validating HDAC2 binding to SALL4 in M010817 cells while none of the other HDACs tested (HDAC1, HDAC4, HDAC6) could be detected in the SALL4 pull-down by western blotting. Note that the same western blot for SALL4 and HDAC2 (left panel) is presented in main Figure 5a but presented here again for reasons of completeness. Source data are provided as Source Data file. Experiment has been repeated independently with similar results two times.

Total target sequences = 7124
Total background sequences = 42224
* - possible false positive

Rank	Motif	P-value	log P-pvalue	% of Targets	% of Background	STD(Bg STD)	Best Match/Details
1	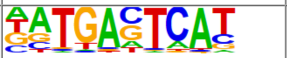	1e-793	-1.828e+03	32.58%	7.77%	102.8bp (86.0bp)	Fra1(bZIP)/BT549-Fra1-ChIP-Seq(GSE46166)/Homer(0.988) More Information | Similar Motifs Found
2	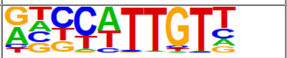	1e-405	-9.336e+02	52.05%	27.78%	107.5bp (86.4bp)	Sox10(HMG)/SciaticNerve-Sox3-ChIP-Seq(GSE35132)/Homer(0.957) More Information | Similar Motifs Found
3	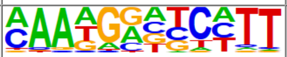	1e-299	-6.893e+02	40.44%	21.05%	109.4bp (83.7bp)	dof4.5/MA1269.1/Jaspar(0.735) More Information | Similar Motifs Found
4	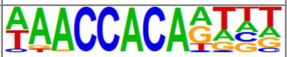	1e-221	-5.092e+02	23.27%	10.23%	101.2bp (84.0bp)	RUNX(Runt)/HPC7-Runx1-ChIP-Seq(GSE22178)/Homer(0.970) More Information | Similar Motifs Found
5	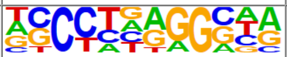	1e-197	-4.551e+02	24.51%	11.67%	106.8bp (76.8bp)	AP-2gamma(AP2)/MCF7-TFAP2C-ChIP-Seq(GSE21234)/Homer(0.916) More Information | Similar Motifs Found
6	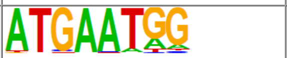	1e-190	-4.395e+02	29.03%	15.23%	107.2bp (90.3bp)	PB0178.1_Sox8_2/Jaspar(0.870) More Information | Similar Motifs Found
7	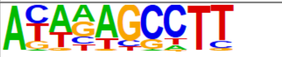	1e-121	-2.800e+02	31.75%	19.94%	109.6bp (84.6bp)	REF2(RRM)/Drosophila_melanogaster-RNCMPT00059-PBM/HughesRNA(0.615) More Information | Similar Motifs Found
8	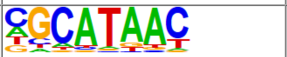	1e-83	-1.920e+02	26.21%	17.04%	116.1bp (86.4bp)	cad/dmmpmm(SeSiMCMC)/fly(0.841) More Information | Similar Motifs Found
9	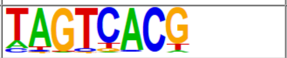	1e-46	-1.060e+02	5.54%	2.48%	101.0bp (88.7bp)	Pax2/MA0067.1/Jaspar(0.833) More Information | Similar Motifs Found
10	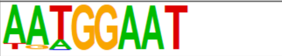	1e-45	-1.046e+02	16.17%	10.63%	111.0bp (87.9bp)	MOD(RRM)/Drosophila_melanogaster-RNCMPT00140-PBM/HughesRNA(0.866) More Information | Similar Motifs Found
11	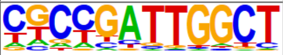	1e-42	-9.797e+01	1.19%	0.17%	97.9bp (85.9bp)	NFY(CCAAT)/Promoter/Homer(0.899) More Information | Similar Motifs Found
12	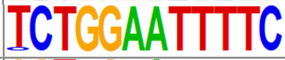	1e-32	-7.588e+01	0.38%	0.01%	76.9bp (75.4bp)	AT2G01818(PLATZ)/col-AT2G01818-DAP-Seq(GSE60143)/Homer(0.820) More Information | Similar Motifs Found
13	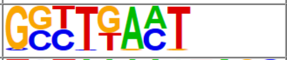	1e-31	-7.173e+01	6.91%	3.93%	120.2bp (86.8bp)	ASH1/Literature(Harbison)/Yeast(0.774) More Information | Similar Motifs Found
14	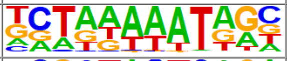	1e-30	-6.940e+01	3.79%	1.73%	103.7bp (85.4bp)	Mef2d(MADS)/Retina-Mef2d-ChIP-Seq(GSE61391)/Homer(0.876) More Information | Similar Motifs Found
15	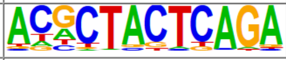	1e-25	-5.933e+01	0.22%	0.00%	86.7bp (65.9bp)	RBM28(RRM)/Homo_sapiens-RNCMPT00049-PBM/HughesRNA(0.681) More Information | Similar Motifs Found
16	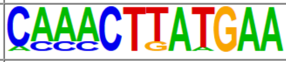	1e-25	-5.772e+01	0.39%	0.02%	52.5bp (64.5bp)	Hoxa13(Homeobox)/ChickenMSG-Hoxa13.Flag-ChIP-Seq(GSE86088)/Homer(0.625) More Information | Similar Motifs Found
17	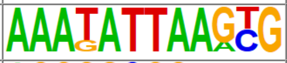	1e-24	-5.530e+01	0.51%	0.05%	102.7bp (72.5bp)	SHEP(RRM)/Drosophila_melanogaster-RNCMPT00068-PBM/HughesRNA(0.727) More Information | Similar Motifs Found
18	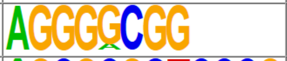	1e-12	-2.910e+01	0.88%	0.30%	81.6bp (77.2bp)	MSN2/MSN2_H2O2Hi/1-MSN2(Harbison)/Yeast(0.954) More Information | Similar Motifs Found
19	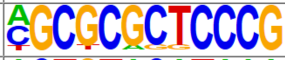	1e-12	-2.830e+01	0.18%	0.01%	57.6bp (46.1bp)	PB0095.1_Zfp161_1/Jaspar(0.754) More Information | Similar Motifs Found
20 *	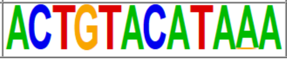	1e-3	-8.321e+00	0.08%	0.01%	90.6bp (80.3bp)	ZBTB32/MA1580.1/Jaspar(0.766) More Information | Similar Motifs Found

Supplementary Figure 8: HOMER *de novo* DNA binding motifs of peaks found with the SALL4 antibody Ab#1 (Abcam, ab29112).

Total target sequences = 9903
Total background sequences = 39621
* - possible false positive

Rank	Motif	P-value	log P-pvalue	% of Targets	% of Background	STD(Bg STD)	Best Match/Details
1	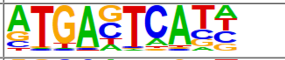	1e-1203	-2.772e+03	31.92%	6.81%	104.7bp (99.5bp)	BATF(bZIP)/Th17-BATF-ChIP-Seq(GSE39756)/Homer(0.989) More Information | Similar Motifs Found
2	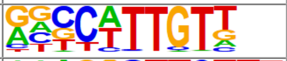	1e-580	-1.338e+03	47.33%	23.51%	115.7bp (99.0bp)	Sox10(HMG)/SciaticNerve-Sox3-ChIP-Seq(GSE35132)/Homer(0.960) More Information | Similar Motifs Found
3	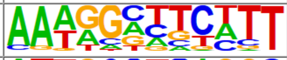	1e-463	-1.066e+03	49.02%	27.19%	116.4bp (96.3bp)	dof4.5/MA1269.1/Jaspar(0.684) More Information | Similar Motifs Found
4	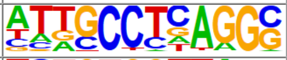	1e-406	-9.359e+02	22.63%	8.38%	115.4bp (90.8bp)	AP-2gamma(AP2)/MCF7-TFAP2C-ChIP-Seq(GSE21234)/Homer(0.940) More Information | Similar Motifs Found
5	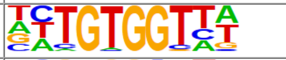	1e-363	-8.368e+02	28.85%	13.20%	120.6bp (97.2bp)	RUNX2(Runt)/PCa-RUNX2-ChIP-Seq(GSE33889)/Homer(0.979) More Information | Similar Motifs Found
6	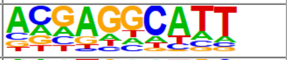	1e-126	-2.911e+02	16.14%	8.64%	128.0bp (97.9bp)	YAP5/MA0417.1/Jaspar(0.712) More Information | Similar Motifs Found
7	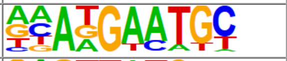	1e-120	-2.772e+02	24.66%	15.57%	124.0bp (102.7bp)	TEC1/TEC1_YPD/[](Harbison)/Yeast(0.842) More Information | Similar Motifs Found
8	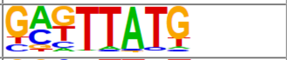	1e-99	-2.289e+02	38.08%	28.20%	158.2bp (98.7bp)	cad/dmmpmm(Bergman)/fly(0.851) More Information | Similar Motifs Found
9	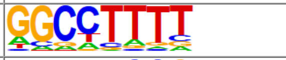	1e-81	-1.884e+02	22.97%	15.57%	127.1bp (96.5bp)	dof42(C2C2dof)/col-dof42-DAP-Seq(GSE60143)/Homer(0.870) More Information | Similar Motifs Found
10	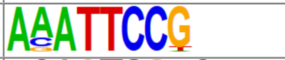	1e-72	-1.681e+02	17.37%	11.22%	126.9bp (98.8bp)	LYS14/MA0325.1/Jaspar(0.913) More Information | Similar Motifs Found
11	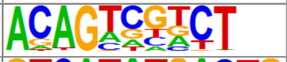	1e-66	-1.524e+02	16.41%	10.69%	142.2bp (99.7bp)	POL009.1_DCE_S_II/Jaspar(0.690) More Information | Similar Motifs Found
12	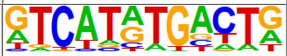	1e-56	-1.301e+02	6.59%	3.35%	121.4bp (102.1bp)	USF1/MA0093.3/Jaspar(0.840) More Information | Similar Motifs Found
13	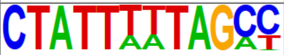	1e-55	-1.282e+02	9.45%	5.49%	156.8bp (94.0bp)	MEF2B/MA0660.1/Jaspar(0.944) More Information | Similar Motifs Found
14	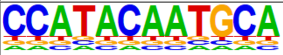	1e-51	-1.181e+02	5.94%	3.00%	119.2bp (100.5bp)	EIL4(EIL)/Tomato-EIL4-ChIP-Seq(GSE116581)/Homer(0.715) More Information | Similar Motifs Found
15	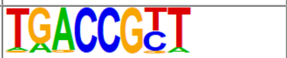	1e-49	-1.149e+02	7.37%	4.08%	118.4bp (96.3bp)	MYB73/MA1394.2/Jaspar(0.926) More Information | Similar Motifs Found
16	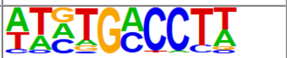	1e-48	-1.120e+02	9.78%	5.97%	120.8bp (95.1bp)	NR2F2/MA1111.1/Jaspar(0.855) More Information | Similar Motifs Found
17	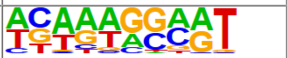	1e-44	-1.026e+02	2.21%	0.72%	97.6bp (99.1bp)	PB0166.1_Sox12_2/Jaspar(0.629) More Information | Similar Motifs Found
18	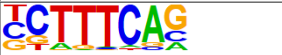	1e-40	-9.434e+01	37.77%	31.38%	133.1bp (101.5bp)	IRF4(IRF)/GM12878-IRF4-ChIP-Seq(GSE32465)/Homer(0.814) More Information | Similar Motifs Found
19	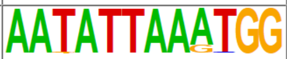	1e-38	-8.766e+01	0.41%	0.02%	85.1bp (62.2bp)	SHEP(RRM)/Drosophila_melanogaster-RNCMPT00068-PBM/HughesRNA(0.734) More Information | Similar Motifs Found
20	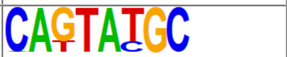	1e-31	-7.166e+01	4.29%	2.32%	125.1bp (103.3bp)	SKO1/MA0382.1/Jaspar(0.649) More Information | Similar Motifs Found
21	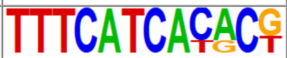	1e-29	-6.729e+01	0.38%	0.03%	98.4bp (57.0bp)	Chop(bZIP)/MEF-Chop-ChIP-Seq(GSE35681)/Homer(0.780) More Information | Similar Motifs Found
22	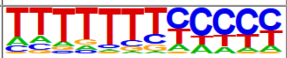	1e-25	-5.835e+01	3.39%	1.82%	121.5bp (86.6bp)	PABPC4(RRM)/Homo_sapiens-RNCMPT00043-PBM/HughesRNA(0.817) More Information | Similar Motifs Found
23	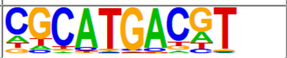	1e-23	-5.473e+01	1.89%	0.81%	123.3bp (100.8bp)	TGA10(bZIP)/colamp-TGA10-DAP-Seq(GSE60143)/Homer(0.835) More Information | Similar Motifs Found
24	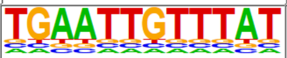	1e-21	-4.946e+01	0.22%	0.01%	80.5bp (73.3bp)	PB0066.1_Sox17_1/Jaspar(0.821) More Information | Similar Motifs Found
25	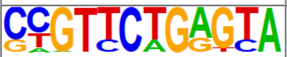	1e-19	-4.520e+01	0.26%	0.02%	91.7bp (50.4bp)	Stat5b/MA1625.1/Jaspar(0.678) More Information | Similar Motifs Found
26	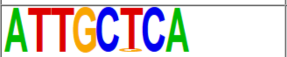	1e-16	-3.810e+01	2.32%	1.27%	117.9bp (97.8bp)	MAC1/MA0326.1/Jaspar(0.802) More Information | Similar Motifs Found

Supplementary Figure 9: HOMER *de novo* DNA binding motifs of peaks found with the SALL4 antibody Ab#2 (Antibodies online, ABIN6132627).

Total target sequences = 4129
Total background sequences = 45055
* - possible false positive

Rank	Motif	P-value	log P-pvalue	% of Targets	% of Background	STD(Bg STD)	Best Match/Details
1	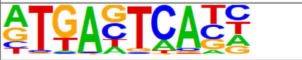	1e-484	-1.116e+03	28.89%	5.68%	52.4bp (63.6bp)	Fra1(bZIP)/BT549-Fra1-ChIP-Seq(GSE46166)/Homer(0.993) More Information | Similar Motifs Found
2	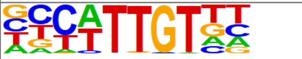	1e-316	-7.294e+02	53.96%	25.99%	52.0bp (61.2bp)	Sox10(HMG)/SciaticNerve-Sox3-ChIP-Seq(GSE35132) /Homer(0.970) More Information | Similar Motifs Found
3	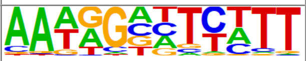	1e-219	-5.053e+02	45.99%	23.45%	51.4bp (58.7bp)	Sox3(HMG)/NPC-Sox3-ChIP-Seq(GSE33059)/Homer(0.563) More Information | Similar Motifs Found
4	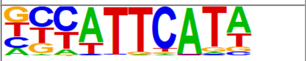	1e-156	-3.596e+02	41.22%	22.58%	52.7bp (64.5bp)	PB0178.1_Sox8_2/Jaspar(0.875) More Information | Similar Motifs Found
5	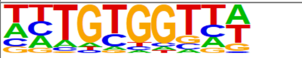	1e-152	-3.512e+02	24.68%	10.30%	52.3bp (59.1bp)	RUNX2(Runt)/PCa-RUNX2-ChIP-Seq(GSE33889)/Homer(0.963) More Information | Similar Motifs Found
6	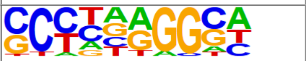	1e-145	-3.355e+02	20.32%	7.68%	55.6bp (60.0bp)	AP-2gamma(AP2)/MCF7-TFAP2C-ChIP-Seq(GSE21234) /Homer(0.879) More Information | Similar Motifs Found
7	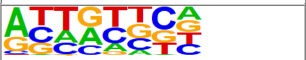	1e-79	-1.830e+02	24.80%	13.72%	54.0bp (64.2bp)	PB0173.1_Sox21_2/Jaspar(0.710) More Information | Similar Motifs Found
8	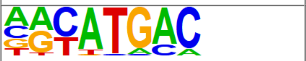	1e-33	-7.751e+01	11.02%	6.02%	57.3bp (67.6bp)	MAFG::NFE2L1/MA0089.1/Jaspar(0.884) More Information | Similar Motifs Found
9	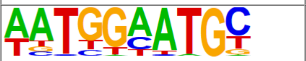	1e-33	-7.701e+01	8.91%	4.50%	56.1bp (65.5bp)	TEAD3(TEA)/HepG2-TEAD3-ChIP-Seq(Encode)/Homer(0.843) More Information | Similar Motifs Found
10	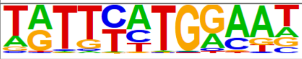	1e-26	-6.161e+01	1.99%	0.45%	51.8bp (61.6bp)	Stat5a::Stat5b/MA0519.1/Jaspar(0.691) More Information | Similar Motifs Found
11	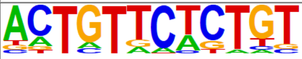	1e-24	-5.584e+01	2.95%	0.99%	56.7bp (62.1bp)	AR-halfsite(NR)/LNCaP-AR-ChIP-Seq(GSE27824)/Homer(0.665) More Information | Similar Motifs Found
12	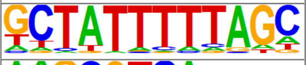	1e-20	-4.816e+01	2.18%	0.66%	50.9bp (57.9bp)	Mef2d(MADS)/Retina-Mef2d-ChIP-Seq(GSE61391)/Homer(0.970) More Information | Similar Motifs Found
13	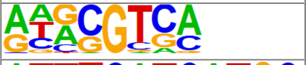	1e-17	-3.976e+01	5.59%	3.04%	57.1bp (62.4bp)	Atf1/MA0604.1/Jaspar(0.815) More Information | Similar Motifs Found
14	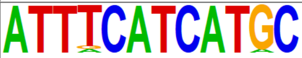	1e-17	-3.961e+01	0.34%	0.01%	41.0bp (9.9bp)	Chop(bZIP)/MEF-Chop-ChIP-Seq(GSE35681)/Homer(0.819) More Information | Similar Motifs Found
15	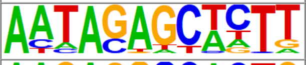	1e-17	-3.941e+01	1.86%	0.59%	55.5bp (58.6bp)	Tcf21/MA0832.1/Jaspar(0.595) More Information | Similar Motifs Found
16	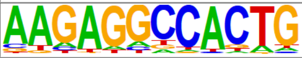	1e-15	-3.501e+01	2.35%	0.92%	58.1bp (63.4bp)	NR4A2/MA0160.1/Jaspar(0.659) More Information | Similar Motifs Found
17	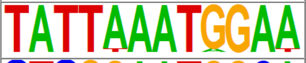	1e-13	-3.177e+01	0.51%	0.05%	49.3bp (54.7bp)	Arid3a/MA0151.1/Jaspar(0.682) More Information | Similar Motifs Found
18	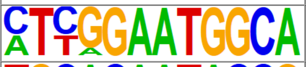	1e-12	-2.828e+01	0.24%	0.01%	57.8bp (28.7bp)	TEAD3/MA0808.1/Jaspar(0.662) More Information | Similar Motifs Found
19	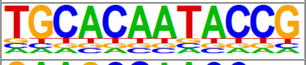	1e-12	-2.825e+01	0.22%	0.01%	47.4bp (43.4bp)	CEBPA/MA0102.3/Jaspar(0.680) More Information | Similar Motifs Found
20 *	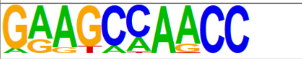	1e-11	-2.707e+01	0.61%	0.10%	56.0bp (64.9bp)	PB0029.1_Hic1_1/Jaspar(0.716) More Information | Similar Motifs Found
21 *	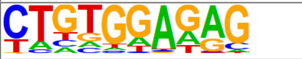	1e-9	-2.257e+01	1.99%	0.91%	57.2bp (60.3bp)	ZNF354C/MA0130.1/Jaspar(0.639) More Information | Similar Motifs Found
22 *	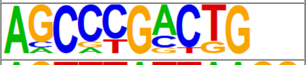	1e-9	-2.140e+01	0.31%	0.03%	63.5bp (57.3bp)	POL006.1_BREu/Jaspar(0.611) More Information | Similar Motifs Found
23 *	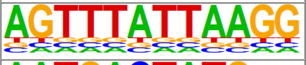	1e-7	-1.692e+01	0.15%	0.01%	49.0bp (10.9bp)	PH0011.1_Alx1_2/Jaspar(0.676) More Information | Similar Motifs Found
24 *	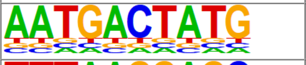	1e-5	-1.176e+01	0.29%	0.06%	35.9bp (15.3bp)	MAFG::NFE2L1/MA0089.1/Jaspar(0.687) More Information | Similar Motifs Found
25 *	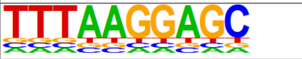	1e-4	-1.060e+01	0.17%	0.02%	63.4bp (32.3bp)	HOXC13/MA0907.1/Jaspar(0.705) More Information | Similar Motifs Found

Supplementary Figure 10: HOMER *de novo* DNA binding motifs of peaks found with the two SALL4 antibodies.

Total target sequences = 3565
Total background sequences = 45111
* - possible false positive

Rank	Motif	P-value	log P-pvalue	% of Targets	% of Background	STD(Bg STD)	Best Match/Details
1	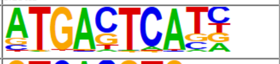	1e-350	-8.061e+02	28.22%	6.49%	86.8bp (85.6bp)	FOSL1/MA0477.2/Jaspar(0.990) More Information | Similar Motifs Found
2	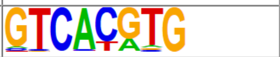	1e-247	-5.691e+02	30.13%	9.86%	85.7bp (90.6bp)	CBF1(MacIsaac)/Yeast(0.976) More Information | Similar Motifs Found
3	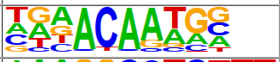	1e-150	-3.458e+02	37.45%	18.71%	89.5bp (81.0bp)	Sox10(HMG)/SciaticNerve-Sox3-ChIP-Seq(GSE35132)/Homer(0.956) More Information | Similar Motifs Found
4	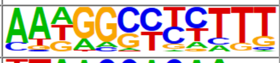	1e-143	-3.303e+02	40.65%	21.62%	86.8bp (79.6bp)	pho/dmmpmm(Bergman)/fly(0.646) More Information | Similar Motifs Found
5	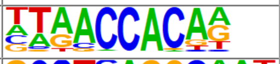	1e-112	-2.592e+02	20.45%	8.28%	96.0bp (81.0bp)	RUNX-AML(Runt)/CD4+-PolII-ChIP-Seq(Barski_et_al.)/Homer(0.957) More Information | Similar Motifs Found
6	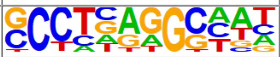	1e-101	-2.346e+02	23.42%	10.78%	92.5bp (84.1bp)	AP-2gamma(AP2)/MCF7-TFAP2C-ChIP-Seq(GSE21234)/Homer(0.951) More Information | Similar Motifs Found
7	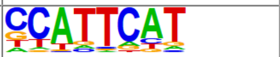	1e-51	-1.180e+02	38.37%	26.72%	90.6bp (84.6bp)	PB0178.1_Sox8_2/Jaspar(0.879) More Information | Similar Motifs Found
8	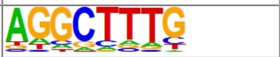	1e-41	-9.644e+01	41.40%	30.58%	97.9bp (84.8bp)	Dof2/MA0020.1/Jaspar(0.805) More Information | Similar Motifs Found
9	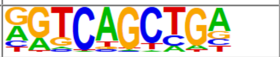	1e-32	-7.520e+01	6.51%	2.68%	99.9bp (91.7bp)	PL0001.1_hlh-11/Jaspar(0.890) More Information | Similar Motifs Found
10	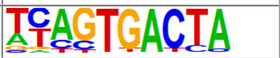	1e-24	-5.673e+01	6.20%	2.87%	85.8bp (81.4bp)	ASH1/Literature(Harbison)/Yeast(0.758) More Information | Similar Motifs Found
11	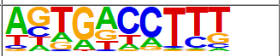	1e-22	-5.102e+01	3.34%	1.17%	97.5bp (83.2bp)	NR4A1/MA1112.2/Jaspar(0.880) More Information | Similar Motifs Found
12	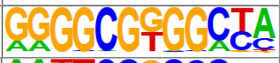	1e-20	-4.685e+01	1.96%	0.50%	98.9bp (72.4bp)	Sp1(Zf)/Promoter/Homer(0.910) More Information | Similar Motifs Found
13	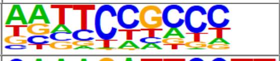	1e-19	-4.420e+01	5.13%	2.44%	91.7bp (80.1bp)	CHA4/MA0283.1/Jaspar(0.766) More Information | Similar Motifs Found
14	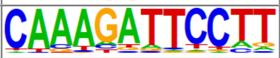	1e-18	-4.350e+01	3.39%	1.33%	87.1bp (78.2bp)	HHO5/MA1164.1/Jaspar(0.780) More Information | Similar Motifs Found
15	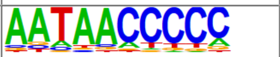	1e-18	-4.177e+01	6.14%	3.22%	90.8bp (75.1bp)	dl/MA0022.1/Jaspar(0.705) More Information | Similar Motifs Found
16	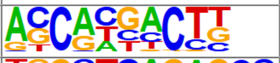	1e-14	-3.394e+01	1.40%	0.36%	87.2bp (87.7bp)	pros/dmmpmm(Bergman)/fly(0.797) More Information | Similar Motifs Found
17	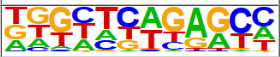	1e-12	-2.956e+01	0.25%	0.01%	89.6bp (10.3bp)	POL010.1_DCE_S_III/Jaspar(0.578) More Information | Similar Motifs Found
18	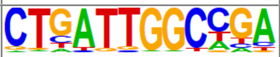	1e-12	-2.955e+01	0.98%	0.21%	62.9bp (85.2bp)	HAP3/MA0314.1/Jaspar(0.868) More Information | Similar Motifs Found
19 *	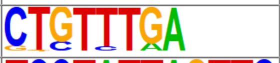	1e-11	-2.720e+01	9.26%	6.23%	94.7bp (85.7bp)	Tv_0258(RRM)/Trichomonas_vaginalis-RNCMPT00258-PBM/HughesRNA(0.797) More Information | Similar Motifs Found
20 *	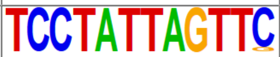	1e-11	-2.692e+01	0.28%	0.01%	42.8bp (24.6bp)	br-Z4/dmmpmm(Pollard)/fly(0.674) More Information | Similar Motifs Found
21 *	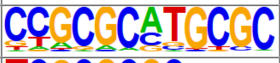	1e-9	-2.296e+01	0.79%	0.17%	76.9bp (72.9bp)	NRF1(NRF)/MCF7-NRF1-ChIP-Seq(Unpublished)/Homer(0.948) More Information | Similar Motifs Found
22 *	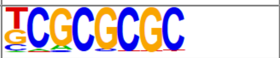	1e-7	-1.813e+01	1.85%	0.85%	90.8bp (87.2bp)	RBM8A(RRM)/Homo_sapiens-RNCMPT00056-PBM/HughesRNA(0.942) More Information | Similar Motifs Found
23 *	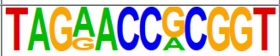	1e-5	-1.339e+01	0.11%	0.00%	50.3bp (0.0bp)	OPI1/MA0349.1/Jaspar(0.615) More Information | Similar Motifs Found

Supplementary Figure 11: HOMER *de novo* DNA binding motifs of peaks found with the HDAC2 antibody Ab#1 (Abcam, ab12169).

Total target sequences = 9819
Total background sequences = 39156
* - possible false positive

Rank	Motif	P-value	log P-pvalue	% of Targets	% of Background	STD(Bg STD)	Best Match/Details
1	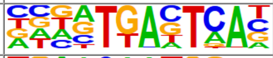	1e-1105	-2.546e+03	32.98%	7.88%	92.6bp (89.1bp)	Fra1(bZIP)/BT549-Fra1-ChIP-Seq(GSE46166)/Homer(0.982) More Information | Similar Motifs Found
2	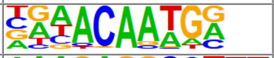	1e-569	-1.311e+03	49.42%	25.35%	99.3bp (85.4bp)	Sox10(HMG)/SciaticNerve-Sox3-ChIP-Seq(GSE35132)/Homer(0.967) More Information | Similar Motifs Found
3	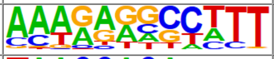	1e-453	-1.044e+03	43.58%	22.76%	98.0bp (81.6bp)	Sox9(HMG)/Limb-SOX9-ChIP-Seq(GSE73225)/Homer(0.659) More Information | Similar Motifs Found
4	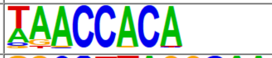	1e-328	-7.558e+02	28.76%	13.71%	102.6bp (86.4bp)	RUNX(Runt)/HPC7-Runx1-ChIP-Seq(GSE22178)/Homer(0.974) More Information | Similar Motifs Found
5	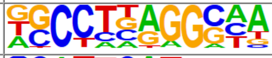	1e-236	-5.456e+02	21.26%	10.00%	97.5bp (89.8bp)	AP-2gamma(AP2)/MCF7-TFAP2C-ChIP-Seq(GSE21234)/Homer(0.950) More Information | Similar Motifs Found
6	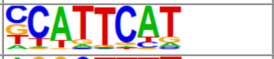	1e-233	-5.369e+02	37.92%	23.20%	102.3bp (89.4bp)	PB0178.1_Sox8_2/Jaspar(0.890) More Information | Similar Motifs Found
7	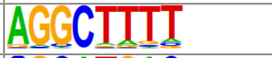	1e-177	-4.096e+02	42.75%	29.20%	108.6bp (87.0bp)	Dof2/MA0020.1/Jaspar(0.781) More Information | Similar Motifs Found
8	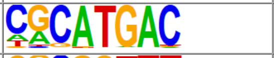	1e-121	-2.807e+02	26.02%	16.62%	106.6bp (93.9bp)	Pax2/MA0067.1/Jaspar(0.828) More Information | Similar Motifs Found
9	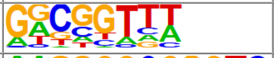	1e-88	-2.039e+02	36.20%	26.96%	108.3bp (87.5bp)	MYB(HTH)/ERMYB-Myb-ChIPSeq(GSE22095)/Homer(0.834) More Information | Similar Motifs Found
10	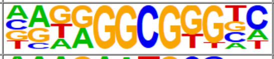	1e-85	-1.959e+02	3.69%	1.08%	96.0bp (74.8bp)	KLF15/MA1513.1/Jaspar(0.884) More Information | Similar Motifs Found
11	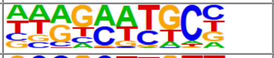	1e-84	-1.954e+02	14.89%	8.80%	106.5bp (91.3bp)	EIF-2ALPHA(S1)/Drosophila_melanogaster-RNCMPT00273-PBM/HughesRNA(0.730) More Information | Similar Motifs Found
12	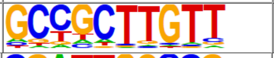	1e-75	-1.729e+02	11.20%	6.24%	96.2bp (91.1bp)	grh/dmmpmm(Bigfoot)/fly(0.713) More Information | Similar Motifs Found
13	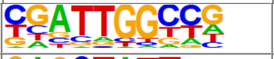	1e-72	-1.659e+02	8.04%	4.01%	101.2bp (88.6bp)	NFY(CCAAT)/Promoter/Homer(0.925) More Information | Similar Motifs Found
14	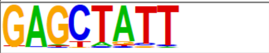	1e-56	-1.294e+02	18.25%	12.63%	104.5bp (87.8bp)	CCA1/MA0972.1/Jaspar(0.696) More Information | Similar Motifs Found
15	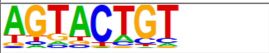	1e-47	-1.103e+02	27.43%	21.21%	106.6bp (88.7bp)	ZBTB32/MA1580.1/Jaspar(0.759) More Information | Similar Motifs Found
16	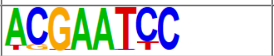	1e-40	-9.272e+01	16.91%	12.27%	111.4bp (87.8bp)	Deaf1/dmmpmm(Pollard)/fly(0.711) More Information | Similar Motifs Found
17	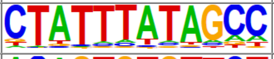	1e-39	-9.013e+01	5.66%	3.10%	95.3bp (75.3bp)	MEF2B/MA0660.1/Jaspar(0.919) More Information | Similar Motifs Found
18	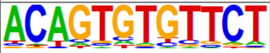	1e-37	-8.586e+01	2.97%	1.27%	100.2bp (83.8bp)	PGR(NR)/EndoStromal-PGR-ChIP-Seq(GSE69539)/Homer(0.812) More Information | Similar Motifs Found
19	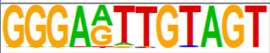	1e-34	-8.027e+01	0.66%	0.08%	85.4bp (83.4bp)	GFY(?)/Promoter/Homer(0.979) More Information | Similar Motifs Found
20	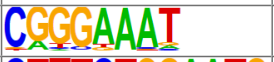	1e-33	-7.812e+01	9.34%	6.16%	115.7bp (84.5bp)	EWS:ERG-fusion(ETS)/CADO_ES1-EWS:ERG-ChIP-Seq(SRA014231)/Homer(0.853) More Information | Similar Motifs Found
21	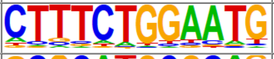	1e-33	-7.754e+01	3.93%	1.99%	99.4bp (90.1bp)	Hsf/MA1458.1/Jaspar(0.791) More Information | Similar Motifs Found
22	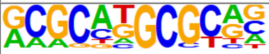	1e-28	-6.576e+01	1.03%	0.27%	91.8bp (98.6bp)	NRF1(NRF)/MCF7-NRF1-ChIP-Seq(Unpublished)/Homer(0.935) More Information | Similar Motifs Found
23	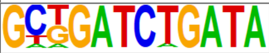	1e-21	-4.889e+01	0.15%	0.00%	47.3bp (48.0bp)	GATA19(C2C2gata)/colamp-GATA19-DAP-Seq(GSE60143)/Homer(0.830) More Information | Similar Motifs Found
24	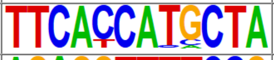	1e-15	-3.624e+01	0.19%	0.02%	94.9bp (45.4bp)	AGL55/MA1202.1/Jaspar(0.704) More Information | Similar Motifs Found
25 *	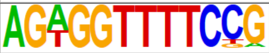	1e-3	-8.113e+00	0.11%	0.03%	148.3bp (58.5bp)	dl(var.2)/MA0023.1/Jaspar(0.776) More Information | Similar Motifs Found

Supplementary Figure 12: HOMER *de novo* DNA binding motifs of peaks found with the HDAC2 antibody Ab#2 (Cell Signaling, 57156S).

Total target sequences = 1043
Total background sequences = 48005
* - possible false positive

Rank	Motif	P-value	log P-pvalue	% of Targets	% of Background	STD(Bg STD)	Best Match/Details
1	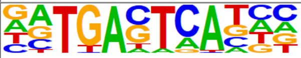	1e-116	-2.690e+02	26.56%	5.00%	51.6bp (65.6bp)	Fra1(bZIP)/BT549-Fra1-ChIP-Seq(GSE46166)/Homer(0.993) More Information | Similar Motifs Found
2	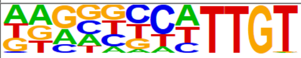	1e-95	-2.209e+02	32.41%	9.20%	51.0bp (63.3bp)	Sox9(HMG)/Limb-SOX9-ChIP-Seq(GSE73225)/Homer(0.946) More Information | Similar Motifs Found
3	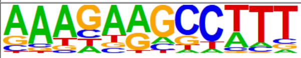	1e-53	-1.230e+02	40.75%	19.79%	52.6bp (61.9bp)	PROX1/MA0794.1/Jaspar(0.625) More Information | Similar Motifs Found
4	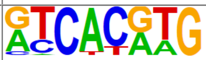	1e-47	-1.103e+02	29.34%	12.28%	54.1bp (66.6bp)	TFE3/MA0831.2/Jaspar(0.962) More Information | Similar Motifs Found
5	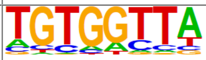	1e-34	-7.902e+01	24.54%	10.97%	55.2bp (64.1bp)	RUNX(Runt)/HPC7-Runx1-ChIP-Seq(GSE22178)/Homer(0.928) More Information | Similar Motifs Found
6	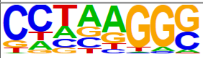	1e-31	-7.150e+01	47.94%	30.59%	53.7bp (62.2bp)	AP-2gamma(AP2)/MCF7-TFAP2C-ChIP-Seq(GSE21234) /Homer(0.856) More Information | Similar Motifs Found
7	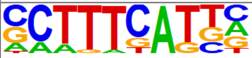	1e-29	-6.752e+01	19.75%	8.46%	51.4bp (64.2bp)	Hoxd10(Homeobox)/ChickenMSG-Hoxd10.Flag-ChIP-Seq(GSE86088) /Homer(0.824) More Information | Similar Motifs Found
8	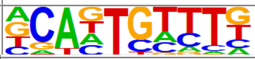	1e-23	-5.409e+01	57.91%	42.31%	56.5bp (64.4bp)	Sox17(HMG)/Endoderm-Sox17-ChIP-Seq(GSE61475)/Homer(0.759) More Information | Similar Motifs Found
9	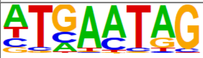	1e-18	-4.370e+01	22.91%	12.72%	53.8bp (64.5bp)	PB0178.1_Sox8_2/Jaspar(0.730) More Information | Similar Motifs Found
10	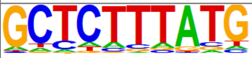	1e-18	-4.272e+01	7.77%	2.43%	54.9bp (61.1bp)	PH0152.1_Pou6f1_2/Jaspar(0.711) More Information | Similar Motifs Found
11	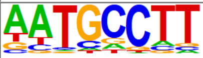	1e-16	-3.764e+01	40.36%	28.31%	56.4bp (65.2bp)	ZNF528(Zf)/HEK293-ZNF528.GFP-ChIP-Seq(GSE58341) /Homer(0.730) More Information | Similar Motifs Found
12	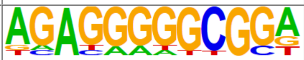	1e-16	-3.712e+01	2.21%	0.20%	60.6bp (54.6bp)	PB0076.1_Sp4_1/Jaspar(0.818) More Information | Similar Motifs Found
13	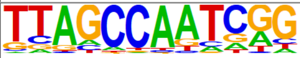	1e-15	-3.622e+01	6.33%	1.93%	51.5bp (63.3bp)	NFY(CCAAT)/Promoter/Homer(0.868) More Information | Similar Motifs Found
14	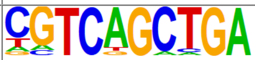	1e-13	-3.145e+01	2.78%	0.45%	59.2bp (68.0bp)	Ap4(bHLH)/AML-Tfap4-ChIP-Seq(GSE45738)/Homer(0.791) More Information | Similar Motifs Found
15	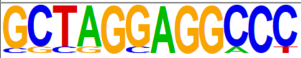	1e-12	-2.772e+01	0.67%	0.01%	57.1bp (52.6bp)	Zac1(Zf)/Neuro2A-Plagl1-ChIP-Seq(GSE75942)/Homer(0.702) More Information | Similar Motifs Found
16 *	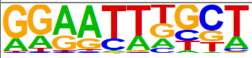	1e-11	-2.729e+01	3.55%	0.86%	56.7bp (63.1bp)	NFkB-p65-Rel(RHD)/ThioMac-LPS-Expression(GSE23622) /Homer(0.718) More Information | Similar Motifs Found
17 *	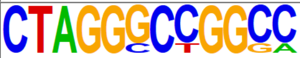	1e-10	-2.396e+01	0.48%	0.00%	41.2bp (0.0bp)	Zfx/MA0146.2/Jaspar(0.618) More Information | Similar Motifs Found
18 *	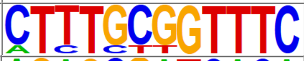	1e-10	-2.303e+01	0.58%	0.01%	59.9bp (33.2bp)	RUNX3/MA0684.1/Jaspar(0.842) More Information | Similar Motifs Found
19 *	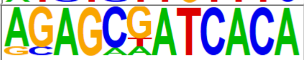	1e-9	-2.292e+01	0.67%	0.01%	57.6bp (37.6bp)	GLI2/MA0734.1/Jaspar(0.632) More Information | Similar Motifs Found
20 *	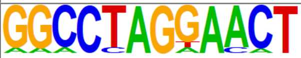	1e-9	-2.213e+01	0.86%	0.04%	58.4bp (45.3bp)	Rfx5(HTH)/GM12878-Rfx5-ChIP-Seq(GSE31477)/Homer(0.702) More Information | Similar Motifs Found
21 *	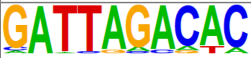	1e-9	-2.175e+01	2.21%	0.43%	51.6bp (70.7bp)	GSC(Homeobox)/FrogEmbryos-GSC-ChIP-Seq(DRA000576) /Homer(0.733) More Information | Similar Motifs Found
22 *	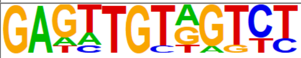	1e-8	-1.853e+01	1.34%	0.18%	60.4bp (62.2bp)	GFY(?)/Promoter/Homer(0.739) More Information | Similar Motifs Found
23 *	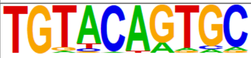	1e-6	-1.414e+01	1.63%	0.37%	43.1bp (67.3bp)	PB0091.1_Zbtb3_1/Jaspar(0.658) More Information | Similar Motifs Found
24 *	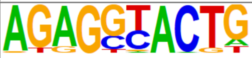	1e-5	-1.333e+01	2.97%	1.12%	52.7bp (66.4bp)	PU.1(ETS)/ThioMac-PU.1-ChIP-Seq(GSE21512)/Homer(0.677) More Information | Similar Motifs Found
25 *	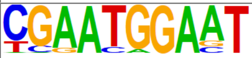	1e-4	-9.987e+00	0.86%	0.16%	49.4bp (55.4bp)	TEAD3(TEA)/HepG2-TEAD3-ChIP-Seq(Encode)/Homer(0.715) More Information | Similar Motifs Found

Supplementary Figures 13: HOMER *de novo* DNA binding motifs of peaks found with the two HDAC2 antibodies.

Total target sequences = 3319
Total background sequences = 45919
* - possible false positive

Rank	Motif	P-value	log P-pvalue	% of Targets	% of Background	STD(Bg STD)	Best Match/Details
1	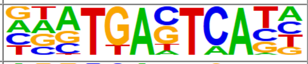	1e-347	-8.002e+02	29.08%	6.49%	53.6bp (65.4bp)	Fra1(bZIP)/BT549-Fra1-ChIP-Seq(GSE46166)/Homer(0.996) More Information | Similar Motifs Found
2	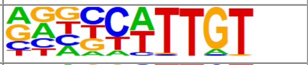	1e-291	-6.721e+02	59.57%	28.99%	52.5bp (62.9bp)	Sox10(HMG)/SciaticNerve-Sox3-ChIP-Seq(GSE35132) /Homer(0.956) More Information | Similar Motifs Found
3	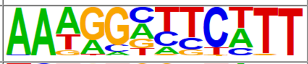	1e-221	-5.100e+02	35.01%	13.25%	51.2bp (59.0bp)	Sox9(HMG)/Limb-SOX9-ChIP-Seq(GSE73225)/Homer(0.604) More Information | Similar Motifs Found
4	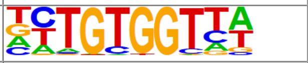	1e-129	-2.973e+02	21.69%	8.10%	52.8bp (61.8bp)	RUNX(Runt)/HPC7-Runx1-ChIP-Seq(GSE22178)/Homer(0.977) More Information | Similar Motifs Found
5	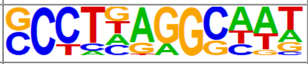	1e-128	-2.954e+02	19.67%	6.89%	53.8bp (60.3bp)	AP-2gamma(AP2)/MCF7-TFAP2C-ChIP-Seq(GSE21234) /Homer(0.930) More Information | Similar Motifs Found
6	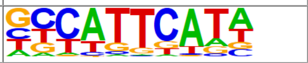	1e-111	-2.576e+02	24.68%	10.80%	52.7bp (67.2bp)	PB0178.1_Sox8_2/Jaspar(0.849) More Information | Similar Motifs Found
7	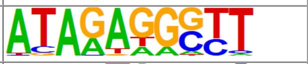	1e-35	-8.255e+01	7.26%	2.91%	54.8bp (58.8bp)	POL012.1_TATA-Box/Jaspar(0.605) More Information | Similar Motifs Found
8	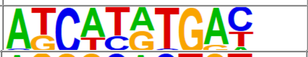	1e-33	-7.671e+01	9.88%	4.78%	58.2bp (64.8bp)	MITF(bHLH)/MastCells-MITF-ChIP-Seq(GSE48085)/Homer(0.870) More Information | Similar Motifs Found
9	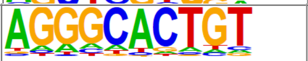	1e-32	-7.463e+01	11.42%	5.93%	56.4bp (62.9bp)	PB0099.1_Zfp691_1/Jaspar(0.708) More Information | Similar Motifs Found
10	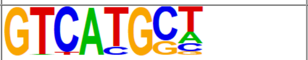	1e-30	-7.111e+01	8.26%	3.81%	56.9bp (65.5bp)	Pax2/MA0067.1/Jaspar(0.873) More Information | Similar Motifs Found
11	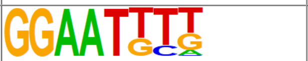	1e-30	-6.970e+01	19.49%	12.42%	56.5bp (63.9bp)	TEAD4/MA0809.1/Jaspar(0.920) More Information | Similar Motifs Found
12	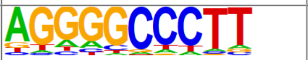	1e-28	-6.501e+01	8.62%	4.21%	54.2bp (62.0bp)	Zac1(Zf)/Neuro2A-Plagl1-ChIP-Seq(GSE75942)/Homer(0.704) More Information | Similar Motifs Found
13	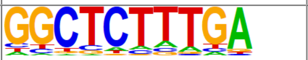	1e-26	-6.126e+01	6.78%	3.05%	55.4bp (63.5bp)	PB0040.1_Lef1_1/Jaspar(0.687) More Information | Similar Motifs Found
14	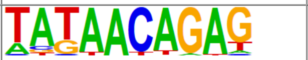	1e-17	-4.014e+01	3.04%	1.13%	50.8bp (63.4bp)	PB0119.1_Foxa2_2/Jaspar(0.714) More Information | Similar Motifs Found
15	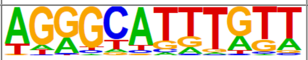	1e-15	-3.547e+01	0.60%	0.05%	53.8bp (73.3bp)	NEUROG2/MA0669.1/Jaspar(0.647) More Information | Similar Motifs Found
16	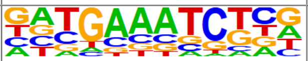	1e-14	-3.255e+01	0.36%	0.01%	40.3bp (16.7bp)	PB0126.1_Gata5_2/Jaspar(0.731) More Information | Similar Motifs Found
17	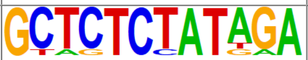	1e-13	-3.059e+01	0.30%	0.01%	68.2bp (17.6bp)	MEF2D/MA0773.1/Jaspar(0.600) More Information | Similar Motifs Found
18 *	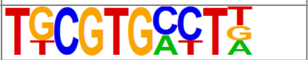	1e-11	-2.621e+01	0.60%	0.08%	52.2bp (61.6bp)	Arnt:Ahr(bHLH)/MCF7-Arnt-ChIP-Seq(Lo_et_al.)/Homer(0.849) More Information | Similar Motifs Found
19 *	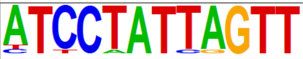	1e-10	-2.347e+01	0.42%	0.04%	60.8bp (32.7bp)	PB0181.1_Spdef_2/Jaspar(0.710) More Information | Similar Motifs Found
20 *	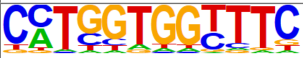	1e-10	-2.303e+01	0.24%	0.01%	47.5bp (61.3bp)	RUNX1(Runt)/Jurkat-RUNX1-ChIP-Seq(GSE29180)/Homer(0.666) More Information | Similar Motifs Found
21 *	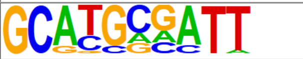	1e-8	-2.008e+01	0.99%	0.29%	53.5bp (63.7bp)	Arnt:Ahr(bHLH)/MCF7-Arnt-ChIP-Seq(Lo_et_al.)/Homer(0.630) More Information | Similar Motifs Found
22 *	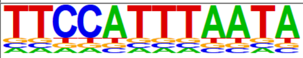	1e-8	-1.942e+01	0.21%	0.01%	55.4bp (38.8bp)	Arid3a/MA0151.1/Jaspar(0.679) More Information | Similar Motifs Found
23 *	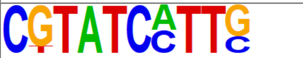	1e-7	-1.721e+01	0.42%	0.06%	50.8bp (58.4bp)	SD0003.1_at_AC_acceptor/Jaspar(0.688) More Information | Similar Motifs Found
24 *	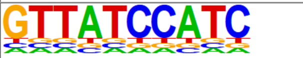	1e-6	-1.594e+01	0.18%	0.01%	53.4bp (55.9bp)	HOXA2(Homeobox)/mES-Hoxa2-ChIP-Seq(Donaldson_et_al.) /Homer(0.738) More Information | Similar Motifs Found

Supplementary Figure 14: HOMER *de novo* DNA binding motifs found in peaks of at least 3 of 4 antibodies (= SALL4-HDAC2 shared motifs)

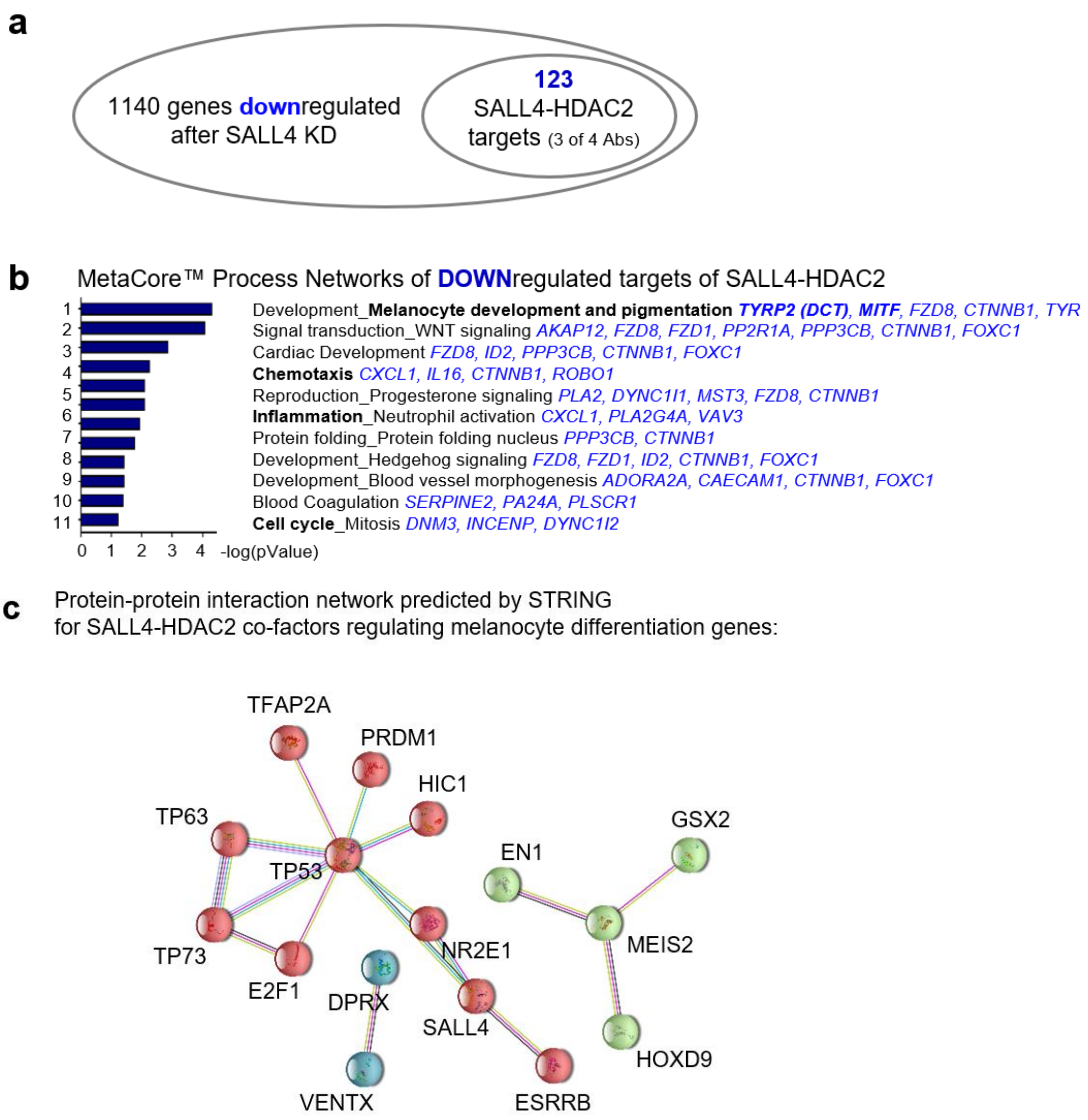

Supplementary Figure 15: SALL4-HDAC2 targets that are downregulated after SALL4 depletion enrich in melanocyte differentiation-related processes. **a** Significantly downregulated genes after SALL4 KD (Figure 4a, c; Supplementary Data 2) were overlaid with the C&R data of direct SALL4-HDAC2 targets (peaks in at least 3 of 4 antibodies) (Figure 5c; Supplementary Data 4), which resulted in 123 direct SALL4-HDAC2 targets significantly downregulated after SALL4 KD (Supplementary Data 4). **b** MetaCore™ Process Network enrichment of the 123 downregulated direct targets from (a) resulted in 11 significant processes (Supplementary Data 4), which are listed with the differentially expressed genes (after SALL4 knock down) of each process. **c** Minimum 3 of 4 C&R peaks in genes related to melanocyte differentiation (Supplementary Data 5) were re-analyzed with CiIIder (Gearing et al., 2019) for transcription factor (TF) enrichments. This resulted in putatively enriched TFs (Supplementary Data 5), which were cut-off for p-values <0.05 and ranked according to significance score (Supplementary Data 5) and further analyzed with STRING (Snel et al., 2000; Szklarczyk et al., 2019) (with strong confidence (0.600) and MCC clustering on default and with hiding unconnected nodes) for putative protein-protein interaction partners of SALL4. This led to the *in silico* identification of a network of 9 putative interaction partners (cluster of red dots/genes) of SALL4 at the analyzed loci of melanocyte differentiation genes.

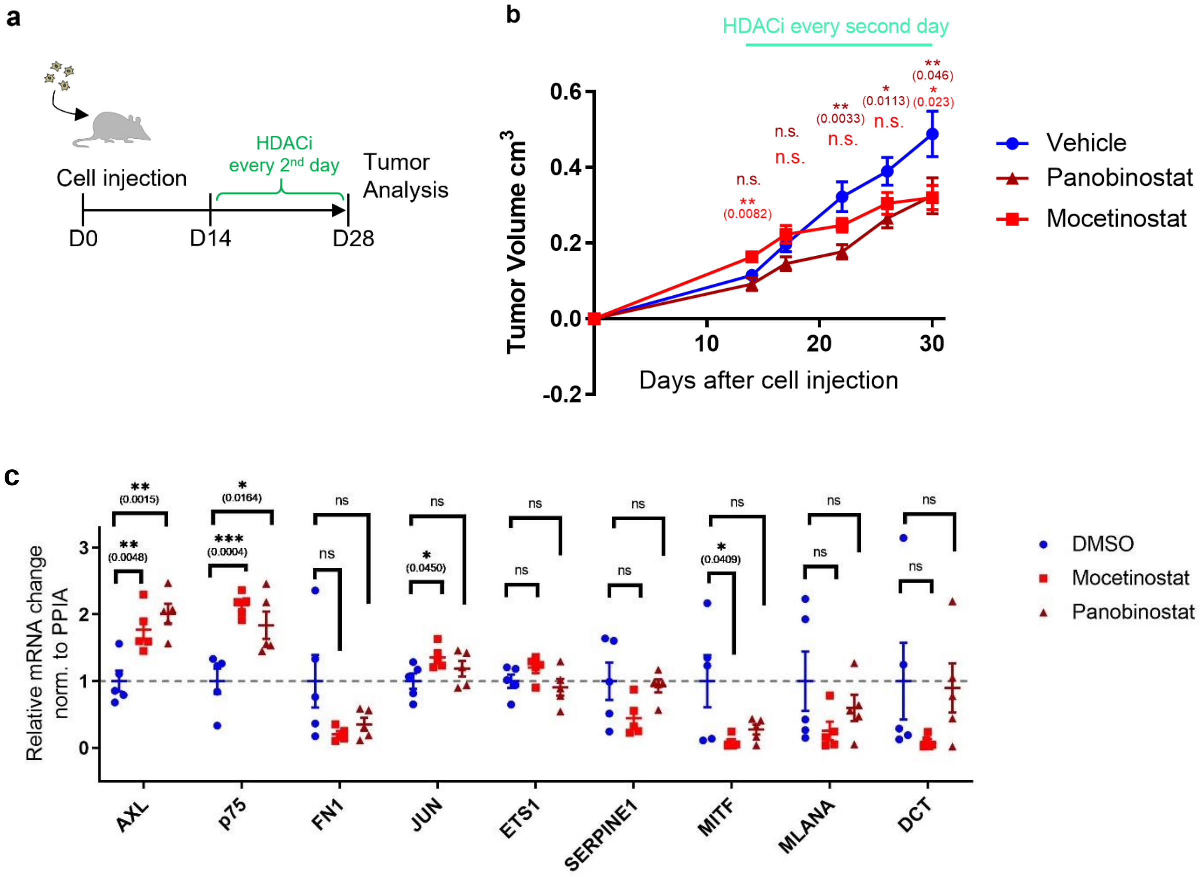

Supplementary Figure 16: *In vivo* HDACi treatment leads to reduced tumor growth and upregulation of invasiveness genes. **a** Setup of *in vivo* HDAC inhibitor treatment of melanoma xenografts. 300’000 M010817 cells were grafted subcutaneously and let grown for 14 days. Then, mice were treated with injections of 10 mg kg^{-1} body weight Panobinostat or 40 mg kg^{-1} body weight Mocetinostat or vehicle every second day for 2 more weeks. **b** Tumor growth curve of (a). **c** qRT-PCR was performed on mRNA extracted from lysed tumors as depicted in (a) at the experimental end point. Gene expression was normalized to PPIA and vehicle-treated samples were set to 1. Error bars represent mean ± SEM in (b) and mean ± SD in (c). For significance, two-sided t-tests were performed with N = 12 (two tumors on a total of 6 mice per group) in (b) and N = 5 in (c) and p-values ≥ 0.05 = n.s.; < 0.05 = *; < 0.01 = ** and < 0.001 = *** and source data for (b) and (c) are provided as Source Data file.

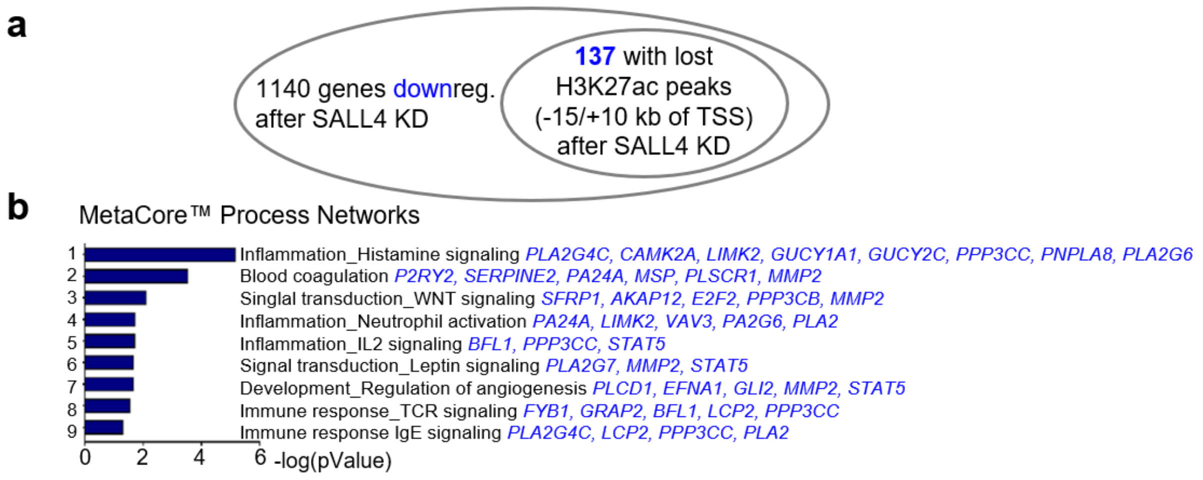

Supplementary Figure 17: Genes that are downregulated and present with lost H3K27ac marks after SALL4 KD. **a** Significantly downregulated genes after SALL4 KD (Figure 4a, c) were overlaid with those genes that have significantly lost H3K27ac marks -15/+10 kb of TSS (Figure 7a, right panel), which resulted in 137 genes with deactivating chromatin marks that are downregulated after SALL4 KD (Supplementary Data 7). **b** MetaCore™ Process Network enrichment of the 137 genes from (a) resulted in 9 significant processes (Supplementary Data 7), which are listed with their DE genes for each process.

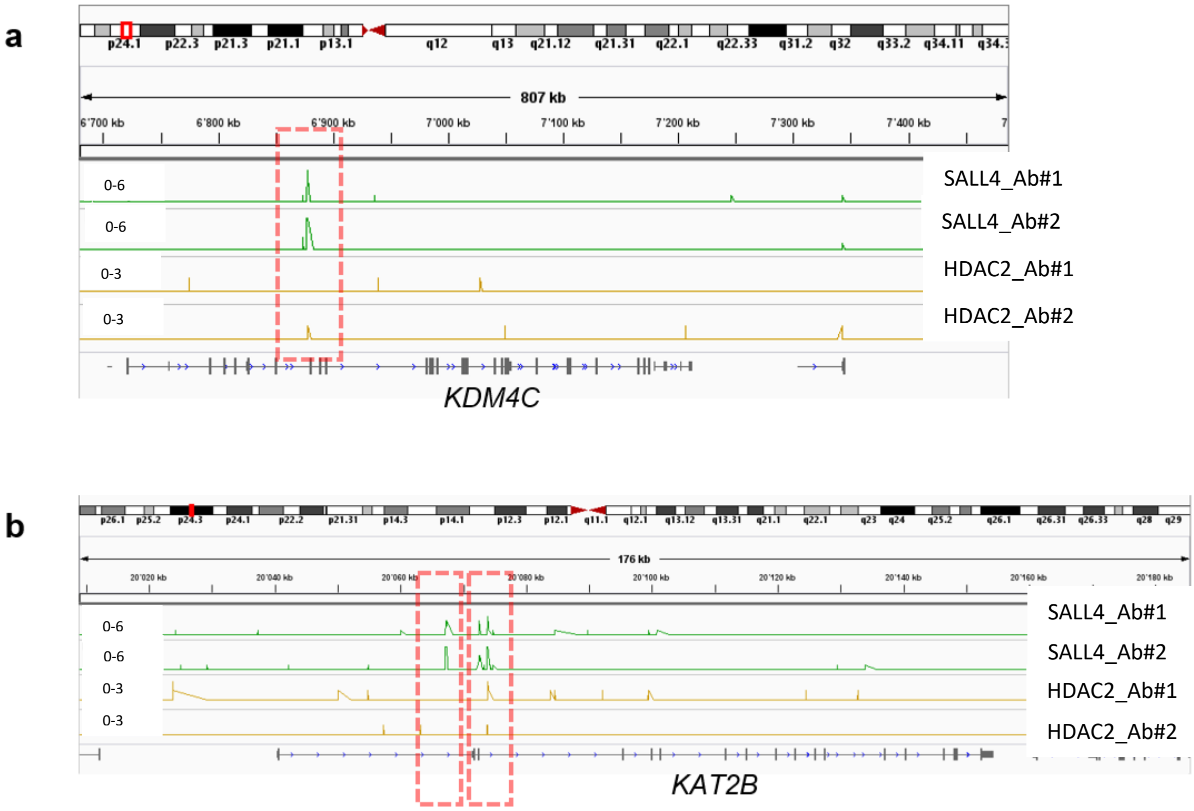

Supplementary Figure 18: CUT&RUN peaks visualized by the Integrative Genome Viewer (IGV) at loci within the histone demethylase *KDM4C* (a) and the histone acetylase *KAT2B* (b) show that both are direct targets of SALL4-HDAC2 (with at least 3 of 4 antibodies) (see also Supplementary Data 4).

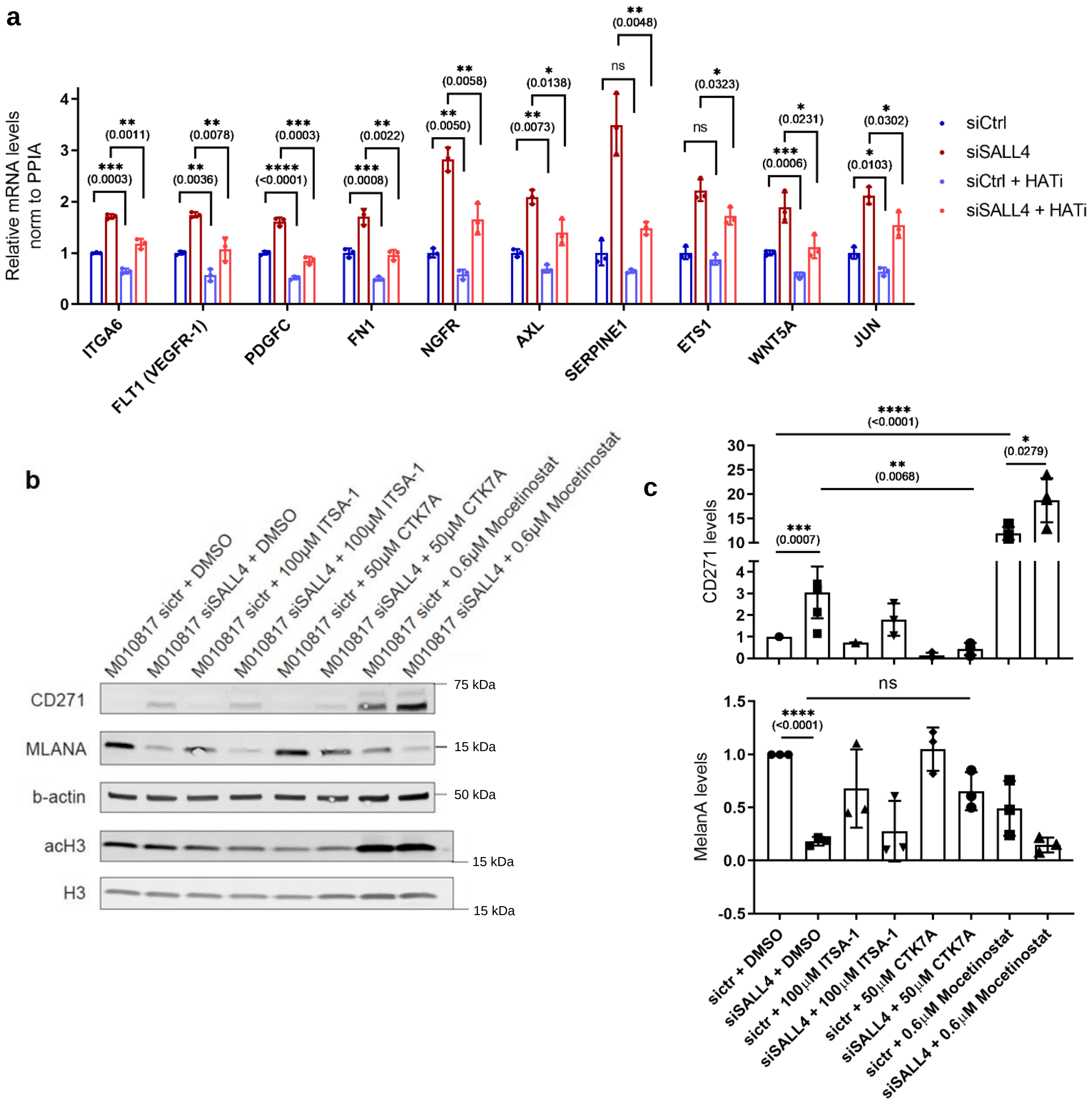

Supplementary Figure 19: a qRT-PCR of M010817 cells co-treated with SALL4 knock down and the histone acetyl transferase (HAT) inhibitor (HATi) CTK7A. Cells had been simultaneously transfected with the corresponding siRNA#1 and treated with 50 µM CTK7A and analyzed for expression of specific genes 48 hrs later. Expression of indicated genes was normalized to the expression of PPIA and control samples were set to 1. **b** Western blot of M010817 cells treated for 48 hrs with vehicle (DMSO), the histone deacetylase (HDAC) activator ITSA-1, the histone acetyl transferase (HAT) inhibitor VII CTK7A or the HDAC inhibitor Mocetinostat at the concentrations indicated. **c** Relative quantification of three independent western blots including (b). Error bars represent mean ± SD and for significance, two-sided T-tests were performed with N = 3 (a and c) and p-values ≥ 0.05 = n.s.; < 0.05 = *; < 0.01 = **; < 0.001 = ***; < 0.0001 = **** and source data for all panels are provided as Source Data file.

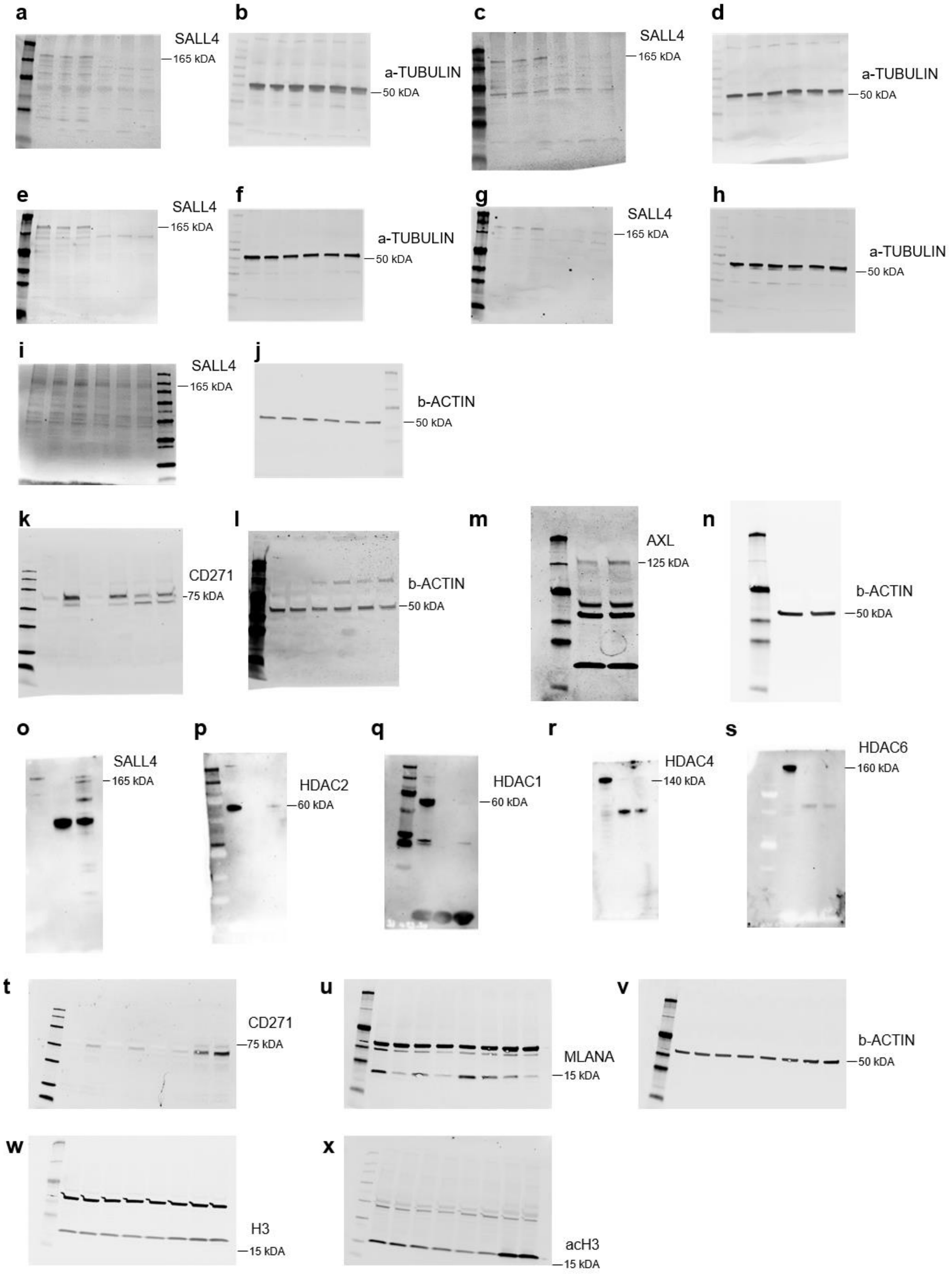

Legend on next page

Supplementary Figure 20: Full blot images of western blots (WBs) presented in the main and Supplementary Figures with indication of the respective Figure and antibody used (see also Supplementary Table 3). **a-b** Full blots of WBs presented in Figure 3a, left panel (a: anti-SALL4 sc-101147; b: anti-a-TUBULIN ab52866). **c-d** Full blots of WBs presented in Figure 3a, right panel (c: anti-SALL4 sc-101147; d: anti-a-TUBULIN ab52866). **e-f** Full blots of WBs presented in Figure 3b, left panel (e: anti-SALL4 sc-101147; f: anti-a-TUBULIN ab52866). **g-h** Full blots of WBs presented in Figure 3b, right panel (g: anti-SALL4 sc-101147; h: anti-a-TUBULIN ab52866). **i-j** Full blots of WBs presented in Figure 3c (i: anti-SALL4 ab29112; j: anti-b-ACTIN ab5316). **k-l** Full blots of WBs presented in Supplementary Figure 6b, top panel (k: anti-CD271 ANT-007-AG; l: anti-b-ACTIN ab5316). **m-n** Full blots of WBs presented in Supplementary Figure 6b, bottom panel (m: anti-AXL sc-1096; n: anti-b-ACTIN ab5316). **o-s** Full blots of WBs presented in Figure 5a (o and p) and Supplementary Figure 7 (all) (o: anti-SALL4 ab29112; p: anti-HDAC2 ab12169; q: anti-HDAC1 #5356; r: anti-HDAC4 #7628; s: anti-HDAC6 #7558). **t-x** Full blots of WBs presented in Supplementary Figure 19b (t: anti-CD271 ANT-007-AG; u: anti-MLANA ab785; v: anti-b-ACTIN ab5316; w: anti-H3 3638S; x: anti-acH3 06-599). See also Source Data.

Supplementary Table 1: Cell lines

Cell Line	Species	Origin	Source	Main Mutation	Treatment
M010817	Human	Skin melanoma metastasis	Dermatology, University Hospital Zurich	*NRAS*^{Q61R}	Radiation; immune therapy with interferon; vaccination with tumour- lysate pulsed DC; refreshing of vaccination; 2x refreshing of vaccination, CR
M070302	Human	Lung melanoma metastasis	Dermatology, University Hospital Zurich	Unknown	Pegasys; therapy with Dacarbazin and Thalidomid (6 cycles) ; immune-modulated therapy (3M-study) ; chemotherapy foursome combination with Velcade; chemotherapy VP-scheme (Eldisine, Platinol, Nexavar, Pegasys)
M150548	Human	Brain melanoma metastasis	Dermatology, University Hospital Zurich	*BRAF*^{V600E}	Targeted therapy with LGX818 (Encorafenib) and MEK162
M121224	Human	Thoracic melanoma metastasis	Dermatology, University Hospital Zurich	*BRAF*^{V600E} *Nras*^{Q61K}	Targeted therapy with LGX818 (Encorafenib) treatment; immune therapy with Ipilimumab
MM150536	Human	Brain melanoma metastasis	Dermatology, University Hospital Zurich	*BRAF*^{V600E}	Targeted therapy with LGX818 (Encorafenib) and MEK162
WM1361A	Human	Primary Cutaneous Melanoma	Commercially available	*NRAS*^{Q61R} *PTEN*$^{+/-}$	

Supplementary Table 2: Primers (qRT-PCR and Genotyping)

**Target (gene or allele)**	**Forward 5' → 3'**	**Reverse 5' → 3'**
**Human qRT-PCR primers:**		
SALL4	ACCGAACCAACACATCCATT	TTGGCTCAGAACCCGTAAAG
NGFR	CCTACGGCTACTACCAGGATG	CACACGGTGTTCTGCTTGT
AXL	GAGGATGAACAGGATGACTGG	ACGAAGGTCTGATGTCCCAGA
SERPINE1	AGTGGACTTTTCAGAGGTGGA	GCCGTTGAAGTAGAGGGCATT
JUN	TGAGTGACCGCGACTTTTCA	GAGGGCATCGTCGTAGAAGG
ETS1	GATAGTTGTGATCGCCTCACC	GTCCTCTGAGTCGAAGCTGTC
WNT5A	ACCGCTTTGCCAAGGAGTTCG	GCCTCGTTGTTGTGCAGGTTCAT
ADAM10	TGCTGAATGGATTGTGGCTCAT	AAAGTGCCTGGAAGTGGTTTAG
MAP4K4	ATTGAGCCCAGGCTTCTGTG	CTGAGTTGCTGGACCCTGAG
FN1	CAGTGGGAGACCTCGAGAAG	TCCCTCGGAACATCAGAAAC
CDH2	ACAGTGGCCACCTACAAAGG	CCGAGATGGGGTTGATAATG
MAPK8	TCTGGTATGATCCTTCTGAAGCA	TCCTCCAAGTCCATAACTTCCTT
ITGA6	CACATCTCCTCCCTGAGCAC	TATCTTGCCACCCATCCTTG
PDGFC	GACTCAGGCGGAATCCAACC	CTTGGGCTGTGAATACTTCCATT
FLT1 (VEGFR-1)	ACCGAATGCCACCTCCATG	AGGCCTTGGGTTTGCTGTC
MITF	CAGGCATGAACACACATTCAC	TCCATCAAGCCCAAGATTTC
MLANA	TGTGCCCTGACCCTACAAGA	CCTCTTCAGCCGTGGTGTAA
DCT	CCAATGATCCCATTTTTGTG	AGGCATCTGCAGGAGGATTA
TRPM1	CACCCAGAGCTACCCAACAGA	CGGATATACATGGCTTTATTGGAA
PPIA	TTCATCTGCACTGCCAAGAC	TCGAGTTGTCCACAGTCAGC
ACTB	GGACTTCGAGCAAGAGATGG	AGGAAGGAAGGCTGGAAGAG
**Mouse genotyping primers:**		
$Sall4^{lox}$	CTCCACCAACTCTAGCTGCTAATGGC	ATGGCCTTTGCACATGTGTTCTGGAG
$Sall4^{wt}$	CTCCACCAACTCTAGCTGCTAATGGC	GTTACAGCAATACGGAGATACACAGC
$Ink4a^{-}$	CTATCAGGACATAGCGTTGG	AGTGAGAGTTTGGGGACAGAG
$Ink4a^{wt}$	ATGATGATGGGCAACGTTC	CAAATATCGCACGATGTC
Cre	AGGCTAAGTGCCTTCTCTACAC	ACCAGGTTCGTTCACTCATGG
GFP	CGCACCATCTTCTTCAAGGACGAC	AACTCCAGCAGGACCATGTGATCG
$tdTomato^{LSL}$	CTGTTCCTGTACGGCATGG	GGCATTAAAGCAGCGTATCC
$tdTomato^{null}$	AAGGGAGCTGCAGTGGAGTA	CCGAAAATCTGTGGGAAGTC

Supplementary Table 3: Antibodies (for stainings, WBs, Co-IP, ChIP seq and CUT&RUN)

**Antibody**	**Host**	**Company**	**Cat. No.**	**Application**	**Dilution**
Sall4	Rabbit	Abcam	ab29112	Immunohistochemistry<br>Co-Immunoprecipitation<br>Western Blot (WB)<br>CUT&RUN (‘SALL4_Ab#1’)	1:200<br>20 ug/sample<br>1:150<br>1:100
Sall4	Rabbit	Antibodies Online	ABIN6132627	CUT&RUN (‘SALL4_Ab#2’)	1:100
Sall4	Mouse	Santa Cruz	sc-101147<br>Clone EE-30<br>Lot D2920	WB	1:100
Sox10	Rabbit	Home-made anti-Sox10 Ab		Immunohistochemistry	1:40’000
Ki67	Rat	Biolegend	652402<br>Clone 16A8<br>Lot B173510	Immunohistochemistry	1:100
Dct	Goat	Santa Cruz	sc-10451	Immunohistochemistry	1:200
Mitf	Rabbit	Gift from H. Arnheiter’s lab	(Bharti et al., 2008; Opdecamp et al., 1997)	Immunohistochemistry	1:200
GFP	Chicken	Aves	GFP-1020	Immunohistochemistry	1:400
HDAC1	Mouse	Cell Signaling	#5356	WB	1:200
HDAC2	Mouse	Cell Signaling	#5113	WB	1:200
HDAC4	Rabbit	Cell Signaling	#7628	WB	1:200
HDAC6	Rabbit	Cell Signaling	#7558	WB	1:200
HDAC2	Mouse	Abcam	ab12169<br>Lot GR321 1055-10	Co-IP<br>CUT&RUN (‘HDAC2_Ab#1’)	20 ug/sample<br>1:100
HDAC2 (D6S5P)	Rabbit	Cell Signaling	57156S<br>Ref 10/2019, Lot 1	CUT&RUN (‘HDAC2_Ab#2’)	1:100
FLAG	Mouse	Sigma-Aldrich	F3165<br>Batch Nb. SLBT 6752	CUT&RUN	1:100
IgG	Rabbit	Abcam	ab6709	Co-IP	20 ug/sample
H3K27ac	Rabbit	Abcam	ab4729<br>Lot GR312658-1	Chromatin immunoprecipitation sequencing	
NGFR	Rabbit	Almone Labs	ANT-007-AG	WB<br>Immunocytochemistry	1:250<br>1:200
AXL	Goat	Santa Cruz	sc-1096	WB	1:250
β-actin	Mouse	Sigma	ab5316	WB	1:400
α-tubulin	Rabbit	Abcam	ab52866<br>Clone EP13324<br>Lot GR3241238-9	WB	1:200
MelanA	Mouse	Abcam	ab785<br>Clone A103 Lot 929307	WB	1:150
FN1	Rabbit	Sigma	F3648	Immunocytochemistry	1:150
VIMENTIN	Mouse	Sigma	V2258<br>Clone LN-6<br>Lot 122M4829	Immunocytochemistry	1:150
CDH2	Rabbit	Takara	M142	Immunocytochemistry	1:250
CDH1	Mouse	BD bioscience	610181<br>Clone 36	Immunocytochemistry	1:150
acH3	Rabbit	Millipore	06-599	WB	1:200
H3	Mouse	Cell Signaling	3638S<br>Clone: 96C10<br>Lot 8	WB	1:200

Supplementary Table 4: siRNAs

Name	Description	Company	Cat. No.	Concentration
siSALL4_1	Stealth siRNA	Invitrogen	10620318 – 332670 B12	50 nM
siControl_1	Stealth Control siRNA, Medium GC Duplex #2	Invitrogen	12935112	50 nM
siSALL4_2	Silencer Select siRNA	Invitrogen	4392421 – s531817	50 nM
siControl_2	Silencer Select siRNA, Negative Control #1	Invitrogen	4390843	50 nM

Supplementary References

1) Gearing, L. J., Cumming, H. E., Chapman, R., Finkel, A. M., Woodhouse, I. B., Luu, K., Gould, J. A., Forster, S. C., & Hertzog, P. J. CiIIder: A tool for predicting and analysing transcription factor binding sites. *PLoS ONE*, *14*(9), e0215495 (2019).

2) Snel, B., Lehmann, G., Bork, P., & Huynen, M. A. String: A web-server to retrieve and display the repeatedly occurring neighbourhood of a gene. *Nucleic Acids Research*, 28(18), 3442-4 (2000).

3) Szklarczyk, D., Gable, A. L., Lyon, D., Junge, A., Wyder, S., Huerta-Cepas, J., Simonovic, M., Doncheva, N. T., Morris, J. H., Bork, P., Jensen, L. J., & Von Mering, C. STRING v11: Protein-protein association networks with increased coverage, supporting functional discovery in genome-wide experimental datasets. *Nucleic Acids Research*, 47(D1), D607-D613 (2019).

4) Bharti, K., Liu, W., Csermely, T., Bertuzzi, S., & Arnheiter, H. Alternative promoter use in eye development: The complex role and regulation of the transcription factor MITF. *Development*, 135(6), 1169-78 (2008).

5) Opdecamp, K., Nakayama, A., Nguyen, M. T. T., Hodgkinson, C. A., Pavan, W. J., & Arnheiter, H. Melanocyte development in vivo and in neural crest cell cultures: Crucial dependence on the Mitf basic-helix-loop-helix-zipper transcription factor. *Development*, 124(12), 2377-86 (1997).
