# Supplementary material for: Epigenetic control of melanoma cell invasiveness by the stem cell factor SALL4
Source: Nat Commun. 2021 Aug 20;12:5056. doi: 10.1038/s41467-021-25326-8 (PMC8379183; doi:10.1038/s41467-021-25326-8)
Supplement: Supplementary file 3 — Description of Additional Supplementary Files [file 41467_2021_25326_MOESM3_ESM.pdf]

Description of Additional Supplementary Files

File Name: Supplementary Data 1
Description: RNA seq of differentially expressed genes in murine hyperplastic (NrasQ61K Ink4a-/-) versus wild-type melanocytes. Cut-offs: LogFC ≥ 0.58 or ≤ -0.58; p-value < 0.05; FDR < 0.05.

File Name: Supplementary Data 2
Description: RNA seq of differentially expressed genes after 48 hrs SALL4 knock down in M010817 human melanoma cells (siSALL4 over siCtrl). Cut-offs: Log2 ratio ≥ 0.27 or ≤ -0.27; p-value < 0.05; FDR < 0.05.

File Name: Supplementary Data 3
Description: Single antibody (2x SALL4; 2x HDAC2) CUT&RUN (C&R) seq peaks in the human melanoma cell line M010817.

File Name: Supplementary Data 4
Description: CUT&RUN seq peaks with minimum 3 of the 4 ('3of4') SALL4/HDAC2 antibodies (= shared targets of SALL4 and HDAC2) in the human melanoma cell line M010817. Correlation of direct SALL4-HDAC2 targets with differential expression after SALL4 knock down. Full MetaCore Process Network analysis of direct SALL4-HDAC2 targets with differential expression.

File Name: Supplementary Data 5
Description: Dataset of selected melanocyte differentiation genes that are SALL4-HDAC2 targets ('3of4' peaks) but downregulated in RNA Seq of siSALL4. CiIlder output of TF enrichment analysis at the selected loci. CiIlder output dataset filtered for high stringency, which was further used for in silico analysis with STRING to determine putative SALL4 interaction partners at melanocyte differentiation gene loci.

File Name: Supplementary Data 6
Description: CUT&RUN seq peaks with exclusively 2 antibodies against SALL4 or 2 antibodies against HDAC2 in the human melanoma cell line M010817. Lists of protein-coding genes that show peaks with SALL4 antibodies exclusively or with HDAC2 antibodies exclusively. MetaCore Process Network enrichment of protein-coding genes that are only bound by SALL4 or HDAC2 respectively.

File Name: Supplementary Data 7
Description: ChIP Seq peaks for H3K27ac after 48 hrs SALL4 knock down in M010817 human melanoma cells (siCtrl and siSALL4 sample).

File Name: Supplementary Data 8
Description: Differential ChIP Seq peaks of H3K27ac in siSALL4- over siCtrl-treated cells (gained or lost peaks upon SALL4 knock-down). Correlation of gained and lost H3K27ac peaks with differential expression. Full MetaCore Process Network analysis of genes with differential acetylation and expression.

File Name: Supplementary Data 9
Description: 3of4 (SALL4/HDAC2 antibody) CUT&RUN peaks associated with differential expression (RNA seq) and differential acetylation (H3K27ac ChIP seq). Full MetaCore Process Network analysis of direct SALL4-HDAC2 targets with differential acetylation and expression.
