# Supplementary material for: Epigenetic control of melanoma cell invasiveness by the stem cell factor SALL4
Source: Nat Commun. 2021 Aug 20;12:5056. doi: 10.1038/s41467-021-25326-8 (PMC8379183; doi:10.1038/s41467-021-25326-8)
Supplement: Supplementary file 13 — Reporting Summary [file 41467_2021_25326_MOESM13_ESM.pdf]

nature research

Corresponding author(s): Lukas Sommer, Johanna Diener

Last updated by author(s): Jun 28, 2021

Reporting Summary

Nature Research wishes to improve the reproducibility of the work that we publish. This form provides structure for consistency and transparency in reporting. For further information on Nature Research policies, see our Editorial Policies and the Editorial Policy Checklist.

Statistics

For all statistical analyses, confirm that the following items are present in the figure legend, table legend, main text, or Methods section.

n/a	Confirmed	
☐	☒	The exact sample size (*n*) for each experimental group/condition, given as a discrete number and unit of measurement
☐	☒	A statement on whether measurements were taken from distinct samples or whether the same sample was measured repeatedly
☐	☒	The statistical test(s) used AND whether they are one- or two-sided<br>*Only common tests should be described solely by name; describe more complex techniques in the Methods section.*
☒	☐	A description of all covariates tested
☒	☐	A description of any assumptions or corrections, such as tests of normality and adjustment for multiple comparisons
☐	☒	A full description of the statistical parameters including central tendency (e.g. means) or other basic estimates (e.g. regression coefficient) AND variation (e.g. standard deviation) or associated estimates of uncertainty (e.g. confidence intervals)
☐	☒	For null hypothesis testing, the test statistic (e.g. *F*, *t*, *r*) with confidence intervals, effect sizes, degrees of freedom and *P* value noted<br>*Give P values as exact values whenever suitable.*
☒	☐	For Bayesian analysis, information on the choice of priors and Markov chain Monte Carlo settings
☒	☐	For hierarchical and complex designs, identification of the appropriate level for tests and full reporting of outcomes
☒	☐	Estimates of effect sizes (e.g. Cohen's *d*, Pearson's *r*), indicating how they were calculated

Our web collection on statistics for biologists contains articles on many of the points above.

Software and code

Policy information about availability of computer code

Data collection	RNA seq, count quantifier and mapping: STAR aligner<br>ChIP seq, mapping of sequence reads: http://bowtie-bio.sourceforge.net/bowtie2/index.shtml<br>CUT&RUN, mapping sequence reads: http://bowtie-bio.sourceforge.net/bowtie2/index.shtml
Data analysis	Analysis of RNA Seq of murine wild-tpe and hyperplastic melanocytes: identification of DE genes with edgeR version v.3.14.0 (http://bioconductor.org/packages/release/bioc/html/edgeR.html)<br>Analysis of RNA seq of SALL4 knock down in human melanoma cells: identification of DE genes with DeSeq2 version 1.16.1 (https://bioconductor.org/packages/release/bioc/html/DESeq2.html)<br>Analysis of ChIP seq of H3K27ac in SALL4 knock down: peak calling with HOMER version 4.11 (http://homer.ucsd.edu/homer/)<br>Analysis of CUT&RUN seq of SALL4 and HDAC2: peak calling with SEACR version 1.3 (https://seacr.fredhutch.org/), DNA binding motif analysis with HOMER version 4.11<br>Analysis of transcription factor enrichment at specific CUT&RUN loci: CiIder (http://ciiider.com/)<br>Single track visualization of ChIP seq and CUT&RUN peaks: Integrative Genome Viewer (IGV) version 2.8.13 (https://software.broadinstitute.org/software/igv/download)<br>Gene Set Enrichment Analysis: GSEA version 4.1.0 (https://www.gsea-msigdb.org/gsea/index.jsp)<br>Process Network Analysis and Pathway Enrichment: MetaCoreTM version 21.2 (https://portal.genego.com/cgi/data_manager.cgi)<br>Analysis of Cell Proliferation with xCELLigence real-time cell analyzer: RTCA Software version 2.1.0<br>Unpaired, two-tailed statistics: GraphPad Prism 5.0 and/or Excel 2019

For manuscripts utilizing custom algorithms or software that are central to the research but not yet described in published literature, software must be made available to editors and reviewers. We strongly encourage code deposition in a community repository (e.g. GitHub). See the Nature Research guidelines for submitting code & software for further information.

Data

Policy information about availability of data

All manuscripts must include a data availability statement. This statement should provide the following information, where applicable:

- Accession codes, unique identifiers, or web links for publicly available datasets
- A list of figures that have associated raw data
- A description of any restrictions on data availability

The datasets generated for this study are available on the European Nucleotide Archive (ENA; https://www.ebi.ac.uk/ena) under accession number PRJEB30285 (RNA Sequencing of hyperplastic and wild-type melanocytes from Varum et al. 2019), PRJEB39208 (RNA Sequencing of siControl and siSALL4-treated M010817 cells), PRJEB39209 (ChIP sequencing of H3K27ac in siControl and siSALL4-treated M010817 cells) and on ArrayExpress (ArrayExpress: https://www.ebi.ac.uk/arrayexpress/) under accession number E-MTAB-10163 (CUT&RUN Sequencing of SALL4 and HDAC2 in M010817 cells) .

Reviewer Access for CUT&RUN data: Username: Reviewer_E-MTAB-10163; Password: r6wFuc5y (dataset will be made publicly available in June 2021)
All other datasets are publicly available already.

Field-specific reporting

Please select the one below that is the best fit for your research. If you are not sure, read the appropriate sections before making your selection.

☒ Life sciences ☐ Behavioural & social sciences ☐ Ecological, evolutionary & environmental sciences

For a reference copy of the document with all sections, see nature.com/documents/nr-reporting-summary-flat.pdf

Life sciences study design

All studies must disclose on these points even when the disclosure is negative.

Sample size

- Experiments with transgenic animals: Previous data from our lab showed that tumors in this spontaneous melanoma model (NrasQ61K Cdkn2a-/-) develop spontaneously with means between 5 to 6 months after birth with a standard deviation of 1 month. In our experiment we wanted to address whether Sall4 conditional knock out in this model affects tumor numbers (and growth) as well as metastasis formation at the experimental endpoint. By calculating at a two-sided significance level of 0.05 the sample size for an estimated effect size of 1.6 times the standard deviation at a power of 0.8, we found that the minimum number of animals needed per experimental group is 16.

- Experiments with immunocompromised mice (human xenografts): In previous experiments with athymic nude mice, we have calculated the smallest possible group size to obtain statistically significant results by nonparametric testing for small sample sizes (<100). We calculated the minimum group size allowing the detection of a true treatment difference of 3 times the standard deviation with a power of 0.8 (at a two-sided 0.05 significance level).

- in vitro Experiments: Experiments were carried out in triplicates and replicated/validated in at least three or more different cell lines. This sample size was chosen based on experience within previous studies and allowed significance calculation on biologically meaningful effect sizes.

Data exclusions

- No data was excluded for in vitro experiments.
- For transgenic animal experiments, data of animals with established, study-independent health issues leading to premature death/euthanasia were excluded from the study.

Replication

Successfull replication of experiments is indicated in the respective figure legends where applicable.

Randomization

- Experiments with transgenic animals: Allocation of animals into groups was not random because the genotypes of the animals were known and determined their allocation towards a specific group (Control, Sall4+/-, Sall4-/-). Whenever possible, we designed our breedings the way that animals of all three genotypes originated from the same litter and hence siblings could be found in all groups (closest genetic background).

- Experiments with immunocompromised animals (human xenografts): Allocation of animals into experimental groups was not randomized to minimize potential non-experiment related biases between animals before onset of the experiment. We allocated animals to the different experimental groups, making sure that a) single cages contained animals of all groups (to avoid cage effects) and b) sample distribution of pre-treatment weight was assessed (if outliers were identified, they were not included in the experiment at all) and animals were allocated to experimental groups resulting with comparable weight means and SDs.

Blinding

Investigators were not blinded for allocation of samples into experimental groups because either genotypes of transgenic animals were known or for xenograft experiments, physiological properties of the mice were assessed before group allocation to avoid non-experiment related physiological biases potentially influencing the experimental outcome. This approach rendered blinded group allocation impossible. For in vitro experiments, no obvious differing covariates that could result in an investigator bias existed between samples.

Reporting for specific materials, systems and methods

We require information from authors about some types of materials, experimental systems and methods used in many studies. Here, indicate whether each material, system or method listed is relevant to your study. If you are not sure if a list item applies to your research, read the appropriate section before selecting a response.

Materials & experimental systems

n/a	Involved in the study
☐	☒ Antibodies
☐	☒ Eukaryotic cell lines
☒	☐ Palaeontology and archaeology
☐	☒ Animals and other organisms
☒	☐ Human research participants
☒	☐ Clinical data
☒	☐ Dual use research of concern

Methods

n/a	Involved in the study
☐	☒ ChIP-seq
☐	☒ Flow cytometry
☒	☐ MRI-based neuroimaging

Antibodies

Antibodies used

All Antibodies used in this study are listed in Supplementary Figures and Tables, Table T3.

Validation

Please see Supplementary Table T3 for product details of each antibody.

-Sall4 ab (Abcam ab29112); own validation (Western blot and immunocytochemistry) and published (e.g. Pantier et al. (2021) and Kong et al. (2021) for C&R and others)
- Sall4 ab (ABIN6132627); own and manufacturer's validation
- Sall4 ab (sc-101147); own validation (Western blot)
- Dct ab (Santa Cruz sc-10451); validated before (Shakhova et al. (2012), Tuncer et al. (2019) and others)
- Sox10 ab (home-made): own validation (Western blot and immunocyto- and -histo chemistry)
- Mitf (Gift from H.Arnheiter) ; validated by others (Bharti et al, (2008) and Opdecamp etal (1997))
- Ki67 ab (652402); own validation and published
- GFP (Aves GFP-1020); own validation (Western blot and immunocytochemistry) on cells transgenically overexpressing GFP
- HDAC1 (#5356); manufacturer's validation and published
- HDAC2 (#5113); own and manufacturer's validation and published
- HDAC2 (ab12169); own and manufacturer's validation and published
- HDAC2 (57156S); own and manufacturer's validation and published
- HDAC4 (#7628); manufacturer's validation
- HDAC6 (#7558); manufacturer's validation
- H3K27ac (Abcam ab4729; Lot GR312658-1); validated by Leone et al. (2017)
- NGFR extracellular (Almone Labs ANT-007-AG); own validation (Western blot and immunocytochemistry) on cells transgenically overexpressing NGFR
- AXL ab (Santa Cruz sc-1096); own validation (wb) and published (Tuncer et al. (2019) and others)
- beta-actin (Sigma ab5316); manufacturer's validation and published
- MelanA (Abcam ab785); manufacturer's validation
- FN1 (Sigma F3648); manufacturer's validation
- Vimentin (Sigma V2258); manufacturer's validation
- CDH2 (Takara M142); manufacturer's validation
- CDH1 (BD biosciences 610181); manufacturer's validation
- acH3 (Millipore 06-599); manufacturer's validation and published
- H3 (Cell Signaling 3638S); manufacturer's validation and published

Eukaryotic cell lines

Policy information about cell lines

Cell line source(s)

See also Supplementary Information.
M010817: Dermatology, University Hospital Zurich
M121224: Dermatology, University Hospital Zurich
MM150536: Dermatology, University Hospital Zurich
M070302: Dermatology, University Hospital Zurich
M150548: Dermatology, University Hospital Zurich
WM1361A: Publicly available (e.g. Rockland Inc.)

Authentication

Authentication by standard procedures by Rockland Inc..

Or according to University Hospital Zurich, Dermatology, where the medical staff at the University Hospital Zurich confirmed the melanoma diagnosis of the tumor material by histology and immunohistochemistry. The selective adherence method of Raaijmakers et al. (2015) was used to establish primary melanoma cell cultures from patient biopsies. The driver mutation of the cell lines (e.g. NRAS; BRAF) was confirmed by PCR. Those primary melanoma cell lines are also included in the URPP biobank, University Hospital Zürich, Department of Dermatology.

Mycoplasma contamination

Cell lines were tested negative for mycoplasma contamination routinely (once a month).

Commonly misidentified lines (See ICLAC register)

No known misidentified cell lines were used in this study.

Animals and other organisms

Policy information about studies involving animals; ARRIVE guidelines recommended for reporting animal research

Laboratory animals	See also Material and Methods section.<br>Tyr::N-RasQ61K animals and Cdkn2a-defficient mice have been described previously (Ackermann et al., 2005; Serrano et al., 1996). Also the Tyr::CreERT2 murine line (Bosenberg et al., 2006), Sall4lox mice (Elling et al., 2006) and R26R-LSL-GFP mice (Simon et al., 2012) have been analyzed previously. Mice were bred and crossed in-house to generate the Tyr::N-RasQ61K; Tyr::CreERT2; Sall4lox/lox; R26R-LSL-GFP genotype and resulted in a mixed genetic background. Mice of both sexes were used for the study and analyzed at the age of 5-6 month of age (98% phenotype (primary tumors) penetrance in the control). For grafting human xenograft cells in vivo, Nude mice (Hsd:AthymicNude-Foxn1nu) were purchased from Harlan and experiments were carried out with female mice of 6–10 weeks of age.
Wild animals	none
Field-collected samples	none
Ethics oversight	All animal experiments have been approved by the veterinary authorities of Canton of Zurich, Switzerland and were performed in accordance with Swiss law.

Note that full information on the approval of the study protocol must also be provided in the manuscript.

ChIP-seq

Data deposition

☒ Confirm that both raw and final processed data have been deposited in a public database such as GEO.

☒ Confirm that you have deposited or provided access to graph files (e.g. BED files) for the called peaks.

Data access links<br>*May remain private before publication.*	Data deposited in European Nucleotide Archive (ENA): https://www.ebi.ac.uk/ena<br>- RNA seq of wild-type vs. Ras Ink4a mutated melanocytes: PRJEB30285<br>- RNA seq of siCtrl vs. siSALL4-treated human melanoma cells (M01): PRJEB39208<br>- ChIP seq of H3K27ac peaks in siCtrl vs. siSALL4-treated human melanoma cells (M01): PRJEB39209<br><br>Data deposited in ArrayExpress (https://www.ebi.ac.uk/arrayexpress/):<br>- CUT&RUN seq of SALL4 and HDAC2 in human melanoma cells (M01): E-MTAB-10163
Files in database submission	- RNA seq of wild-type vs. Ras Ink4a mutated melanocytes: 3x wild-type samples; 3x Ras Ink4a mutated melanocytes samples<br><br>- RNA seq of siCtrl vs. siSALL4-treated human melanoma cells (M01):<br>Sample ERS4803638 (SAMEA7039447) M010817_siCtr_1<br>Sample ERS4803639 (SAMEA7039448) M010817_siCtr_2<br>Sample ERS4803640 (SAMEA7039449) M010817_siCtr_3<br>Sample ERS4803641 (SAMEA7039450) M010817_siSALL4_1<br>Sample ERS4803642 (SAMEA7039451) M010817_siSALL4_2<br>Sample ERS4803643 (SAMEA7039452) M010817_siSALL4_3<br><br>- ChIP seq of H3K27ac peaks in siCtrl vs. siSALL4-treated human melanoma cells (M01):<br>Sample ERS4803644 (SAMEA7039453) siCtrl<br>Sample ERS4803645 (SAMEA7039454) siCtrl_Input<br>Sample ERS4803646 (SAMEA7039455) siSALL4<br>Sample ERS4803647 (SAMEA7039456) siSALL4_Input<br><br>- CUT&RUN seq of SALL4 and HDAC2 in human melanoma cells (M01):<br>Samples Di-F_XXX FLAG antibody<br>Samples Di-H1_XXX HDAC2 antibody 1<br>Samples Di-H2_XXX HDAC2 antibody 2<br>Samples Di-S1_XXX SALL4 antibody 1<br>Samples Di-S2_XXX SALL4 antibody 2
Genome browser session (e.g. UCSC)	no longer applicable

Methodology

Replicates	RNA Seq: 3 experimental replica per group<br>ChIP seq: no replicates (1x siCtrl sample; 1x siSALL4 sample)<br>CUT&RUN: 2 different antibodies per factor

Sequencing depth	Please see Material and Methods section for details of RNA seq, ChIP seq and CUT&RUN seq performed for this study.
Antibodies	ChIP seq:<br>rabbit H3K27ac (Abcam ab4729; Lot GR312658-1)<br>CUT&RUN seq:<br>SALL4 (antibodies ab29112 (Abcam) and ABIN6132627 (Antibodies Online))<br>HDAC2 (antibodies ab12169 (Abcam) and 57156S (Cell Signaling))
Peak calling parameters	Peak calling was performed with the HOMER tool package (http://homer.ucsd.edu/homer/) for ChIP seq of H3K27ac after SALL4 knock down and with SEACR (https://seacr.fredhutch.org/) for CUT&RUN of SALL4 and HDAC2.
Data quality	ChIP seq:<br>H3K27ac peaks (in any genetic region) with FDR < 0.05 and fold enrichment >5:<br>siCtrl: 71'264<br>siSALL4: 74'929<br><br>CUT&RUN seq:<br>SALL4 antibody_1: 7124 peaks<br>SALL4 antibody_2: 9902 peaks<br>HDAC2 antibody_1: 3567 peaks<br>HDAC2 antibody_2: 9818 peaks
Software	ChIP seq, mapping of sequence reads: http://bowtie-bio.sourceforge.net/bowtie2/index.shtml<br>Analysis of ChIP seq, peak calling: http://homer.ucsd.edu/homer/<br>Single track visualization of ChIP seq peaks: https://igv.org/app/<br><br>CUT&RUN seq, mapping of sequence reads: http://bowtie-bio.sourceforge.net/bowtie2/index.shtml<br>Analysis of CUT&RUN seq, peak calling: SEACR (https://seacr.fredhutch.org/)<br>Single track visualization of CUT&RUN seq peaks: https://igv.org/app/

Flow Cytometry

Plots

Confirm that:

☐ The axis labels state the marker and fluorochrome used (e.g. CD4-FITC).

☐ The axis scales are clearly visible. Include numbers along axes only for bottom left plot of group (a 'group' is an analysis of identical markers).

☐ All plots are contour plots with outliers or pseudocolor plots.

☐ A numerical value for number of cells or percentage (with statistics) is provided.

Methodology

Sample preparation	*Describe the sample preparation, detailing the biological source of the cells and any tissue processing steps used.*
Instrument	*Identify the instrument used for data collection, specifying make and model number.*
Software	*Describe the software used to collect and analyze the flow cytometry data. For custom code that has been deposited into a community repository, provide accession details.*
Cell population abundance	*Describe the abundance of the relevant cell populations within post-sort fractions, providing details on the purity of the samples and how it was determined.*
Gating strategy	*Describe the gating strategy used for all relevant experiments, specifying the preliminary FSC/SSC gates of the starting cell population, indicating where boundaries between "positive" and "negative" staining cell populations are defined.*

☐ Tick this box to confirm that a figure exemplifying the gating strategy is provided in the Supplementary Information.
